# A Novel Rubodvirus Associated with Progressive Leaf Whitening Disease in Bougainvillea: Genomic Insights and Phylogenetic Relationships

**DOI:** 10.3390/plants15203193

**Published:** 2026-10-18

**Authors:** Haiyan Che, Ruyu Cai, Daquan Luo, Haibo Long

**Affiliations:** 1 Environment and Plant Protection Institute, Chinese Academy of Tropical Agricultural Sciences, Haikou 571101, China; 16608916899@163.com (R.C.); luodaquan@163.com (D.L.); 2 Hainan Key Laboratory for Monitoring and Control of Tropical Agricultural Pests, Haikou 571101, China

**Keywords:** *Bougainvillea* sp., rubodvirus, high-throughput sequencing, progressive leaf whitening

## Abstract

*Bougainvillea* sp. is an important ornamental and landscape plant in tropical and subtropical regions. In October 2024, progressive leaf whitening resembling viral infection was observed in Haikou, Hainan Province, China. To investigate the factors associated with progressive leaf whitening in bougainvillea (particularly viral pathogens), we performed virome analysis of two symptomatic leaf samples using total RNA sequencing with ribosomal RNA depletion on the Illumina NovaSeq 6000 platform. A novel tripartite negative-sense RNA virus was identified and tentatively named Bougainvillea progressive whitening virus (BgPWV), belonging to the genus *Rubodvirus* in the family *Phenuiviridae*. Its genome comprises RNA1–RNA3, each containing a single open reading frame flanked by untranslated regions, encoding the large protein (302 kDa), movement protein (43 kDa), and nucleocapsid protein (32 kDa), respectively. The 5′ and 3′ terminal sequences of each genomic RNA are highly conserved and complementary. Molecular screening of 54 bougainvillea plants showed that all 14 symptomatic plants tested positive for BgPWV, whereas all 40 asymptomatic samples tested were negative; three symptomatic plants were co-infected with Bougainvillea chlorotic vein banding virus. These results indicate a significant association between BgPWV and leaf whitening and expand the known host range and genetic diversity of rubodviruses, providing a foundation for future studies on bougainvillea viral diseases.

## 1. Introduction

Bougainvillea (*Bougainvillea* sp.), belonging to the family *Nyctaginaceae*, is native to the tropical and subtropical regions of South America and has been widely cultivated worldwide [1,2]. Bougainvillea is prized for its vibrant, long-lasting, and colorful bracts, adaptability to various climatic conditions, and relatively low maintenance requirements, making it an important ornamental and landscaping plant in tropical and subtropical regions. In China, bougainvillea holds particular significance—it is the symbolic flower of Hainan Province and is extensively cultivated in Guangdong, Guangxi, Fujian, Yunnan, and other southern provinces [2,3]. The ornamental horticulture industry around bougainvillea has grown substantially, with numerous cultivars being developed and propagated for commercial production.

Although bougainvillea is distributed worldwide, reports of viral diseases affecting this plant remain relatively scarce. The most extensively studied virus infecting bougainvillea is Bougainvillea chlorotic vein banding virus (BCVBV), also known as Bougainvillea spectabilis chlorotic vein-banding virus (BsCVBV), a member of the genus *Badnavirus* in the family *Caulimoviridae*. This virus was first reported in Brazil in 2001, causing chlorotic vein-banding symptoms, and has since been reported in India, Malaysia, and other countries [3,4,5,6,7,8,9,10,11]. In China, the virus was detected in Taiwan in 2005, where infected plants of the susceptible cultivar “Taipei Red” exhibited mottling, chlorosis, vein banding, stunting, and severe leaf deformation. Electron microscopy revealed typical bacilliform particles approximately 28 × 130–150 nm in size in infected cells [10]. In 2020, it was first reported in Hainan Province, with an incidence exceeding 30% in the surveyed area [3], and in 2023, it was also detected in Guangdong [11]. In addition, another virus associated with chlorotic spots symptoms, Bougainvillea glabra chlorotic spot virus, has been reported in Egypt and also belongs to the genus *Badnavirus*, family *Caulimoviridae* [12].

The advent of high-throughput sequencing (HTS) technologies has revolutionized plant virology by enabling unbiased, large-scale identification of both known and previously uncharacterized viruses directly from plant samples [13,14]. HTS-based approaches have been successfully applied to discover novel viruses in a wide array of ornamental, crop, and wild plant species, significantly expanding our understanding of plant virus diversity and evolution [15,16,17]. In this study, we employed HTS and RT-PCR to identify and genomically characterize a novel virus infecting bougainvillea plants exhibiting progressive leaf whitening. Phylogenetic and sequence identity analyses were performed to determine its taxonomic position and evolutionary relationships with known viruses. Our findings expand the known viral diversity associated with bougainvillea and provide a foundation for future studies on the biology, epidemiology, and management of viral diseases in this important ornamental plant.

## 2. Results

### 2.1. Field Disease Survey

**Figure 1:**
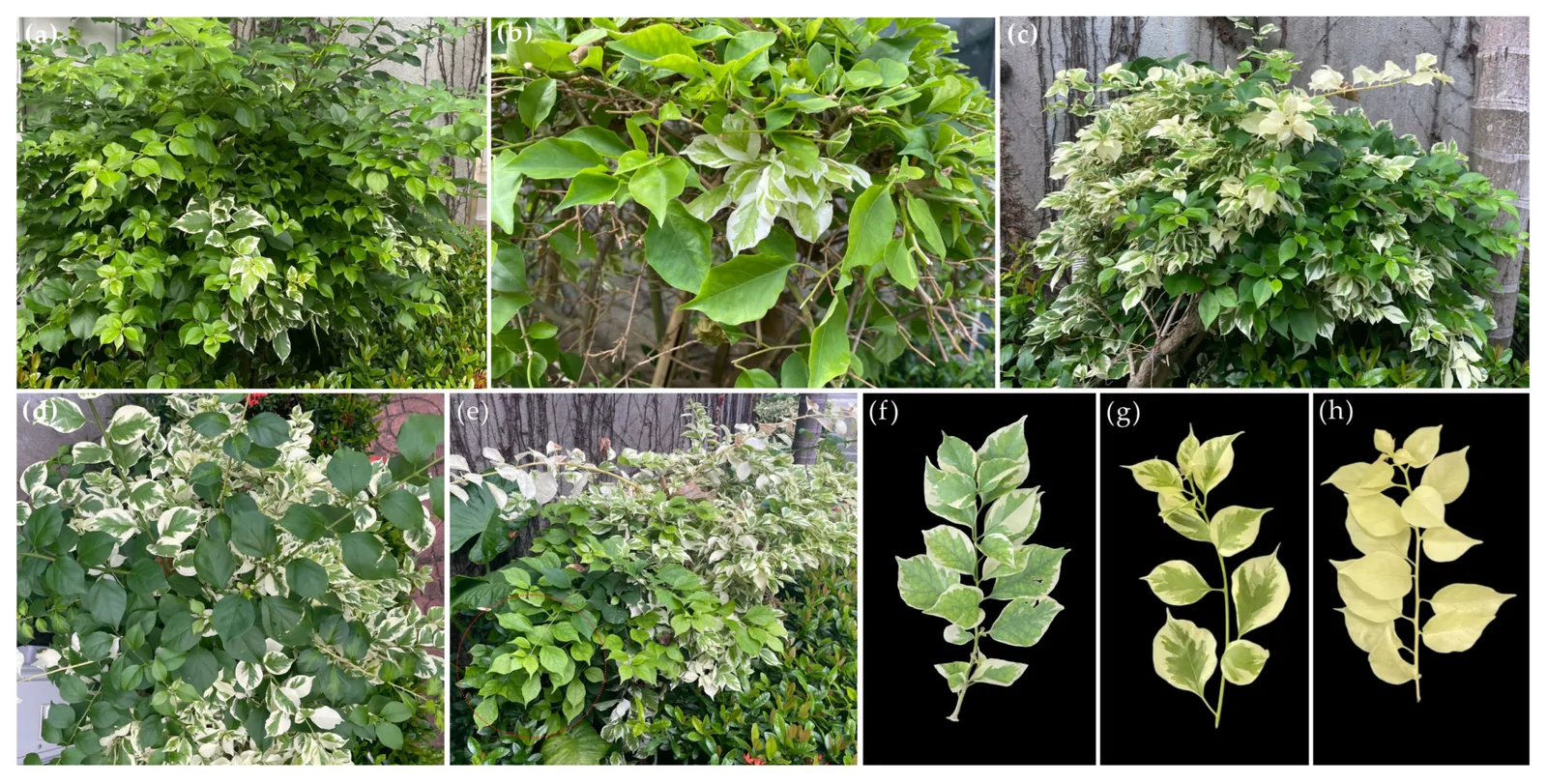
Progressive leaf whitening symptoms in a bougainvillea plant in a greenbelt in Haikou, Hainan Province, China. (**a**,**b**) Creamy-white margins on leaf edges of some branches. (**c**,**d**) Symptoms spreading from a few branches to most branches. (**e**,**f**) Chlorotic vein banding symptoms on leaves of the affected plant. (**g**) Creamy-white areas progressively expanding toward the midrib. (**h**) Complete leaf whitening.

In October 2024, a routine survey of ornamental plant diseases was conducted in a greenbelt in Haikou, Hainan. One bougainvillea plant was observed in a greenbelt, with leaf margins on a few branches exhibiting creamy-white discoloration (Figure 1a,b). Continuous observation revealed that these creamy-white areas progressively expanded toward the midrib (Figure 1g), eventually resulting in complete chlorosis of the leaves (Figure 1h). Over time, the symptoms spread from a few branches to most branches and eventually to the entire plant (Figure 1c,d). By May 2026, all 14 Bougainvillea plants in the same greenbelt had developed the aforementioned symptoms. At the time of sampling, all surveyed plants were in the vegetative growth stage, and no flowering, fruiting, or seed production was observed. To date, no plants with similar symptoms have been observed elsewhere in Hainan. Additionally, three of the 14 affected plants exhibited chlorotic vein banding on the leaves (Figure 1e,f).

### 2.2. HTS Output and Discovery of Phenuiviridae Members in Bougainvillea

Two bougainvillea plants (designated a1 and a2) exhibiting creamy-white leaf margins were collected from Haikou City, Hainan Province, China (Section 4.1). Equal amounts of total RNA from the two plants were pooled as sample PL-1 and subjected to rRNA-depleted total RNA sequencing, generating 75,344,936 raw reads, with Q20 and Q30 base percentages both exceeding 95.39%. After quality control, 72,681,806 clean reads were obtained. Of these, 58,493,500 reads (80.48%) were mapped to the *Bougainvillea glabra* reference genome (GCA_045838725.1), while the remaining 14,188,306 reads (19.52%) that did not map to the host genome were subjected to de novo assembly. The resulting contigs were then subjected to BLASTn and BLASTx analyses using BLAST+ version 2.17.0 analyses against the NCBI non-redundant nucleotide (nt) and protein (nr) databases, respectively.

These analyses revealed that the majority of contigs shared similarity with non-plant-associated viral agents. Ten contigs (ranging from 222 to 7983 nt in length) shared sequence similarity with members of genus *Rubodvirus* in the family *Phenuiviridae*. The proteins encoded by these contigs exhibited 48.6–74.8% amino acid (aa) sequence identity with known rubodviruses (Table 1). The relatively low aa identity with known rubodviruses indicated the existence of a novel member of *Rubodvirus*, which was designated Bougainvillea progressive whitening virus (BgPWV). Notably, no other virus- or viroid-related sequences were identified during the analysis.

To confirm that this putative virus was not an artifact generated by HTS, three pairs of primers (Table S1) were designed based on the assembled contigs. Separate reverse transcription-PCR (RT-PCR) assays were performed using the original plant samples (a1 and a2) used for HTS (Figure S1), and the resulting amplicons were sequenced. Both samples tested positive for BgPWV, and the sequences of the resulting amplicons showed 99–100% identity with the corresponding HTS-derived sequences.

### 2.3. Genomic Organization of the Novel Rubodvirus

The two bougainvillea samples (a1 and a2) that tested positive for BgPWV in Section 2.2 were used for genome reconstruction. Each viral RNA segment was amplified by RT-PCR to cover nearly its full length, and the 5′ and 3′ terminal sequences were determined by RACE, followed by Sanger sequencing (Section 4.2). Two virus isolates, designated a1 (RNA1–RNA3 lengths: 8007, 1322, and 1370 bp; GenBank accessions PZ832366, PZ832368, and PZ832367) and a2 (lengths: 8007, 1322, and 1361 bp; accessions PZ832369, PZ832371, and PZ832370), were obtained. Both isolates possess tripartite genomes of negative-sense RNA. The sequence identities for the corresponding segments (RNA1, RNA2, RNA3) between the two isolates range from 98.36% to 99.02%.

**Figure 2:**
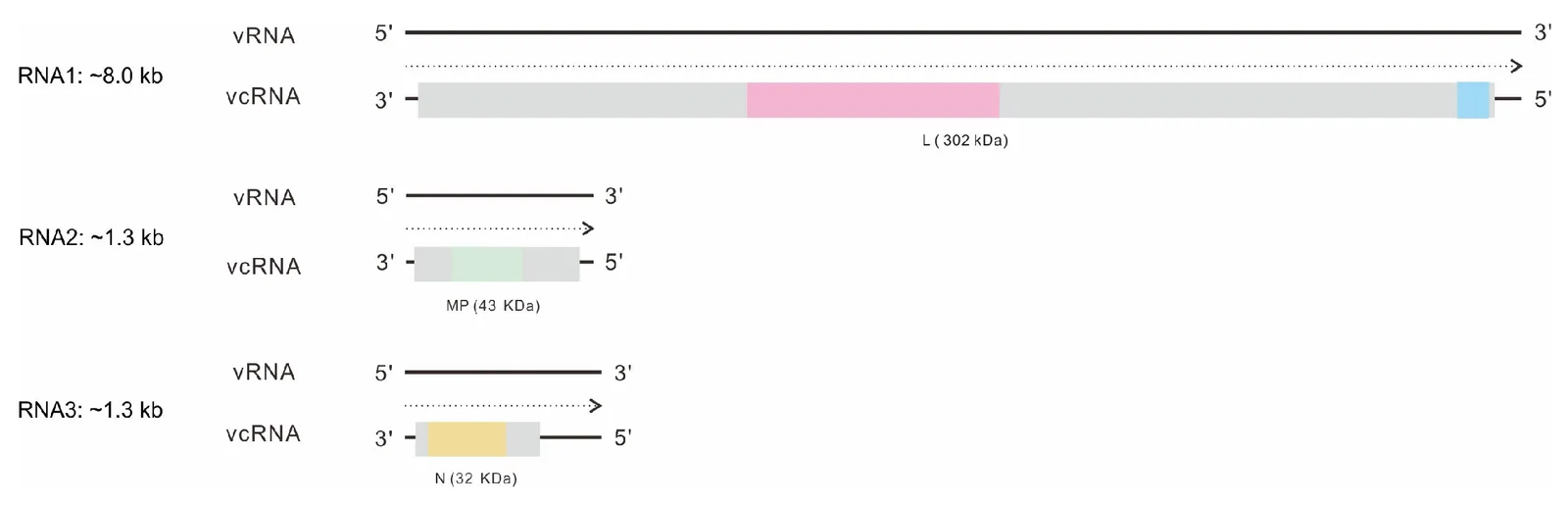
Schematic representation of the genome organization of Bougainvillea progressive whitening virus (BgPWV). vRNA, virion RNA; vcRNA, viral complementary RNA; L, large protein; MP, movement protein; N, nucleocapsid protein; The dashed arrow indicates the ORF orientation on the vcRNA; Conserved motifs are shown in different colors.

**Figure 3:**
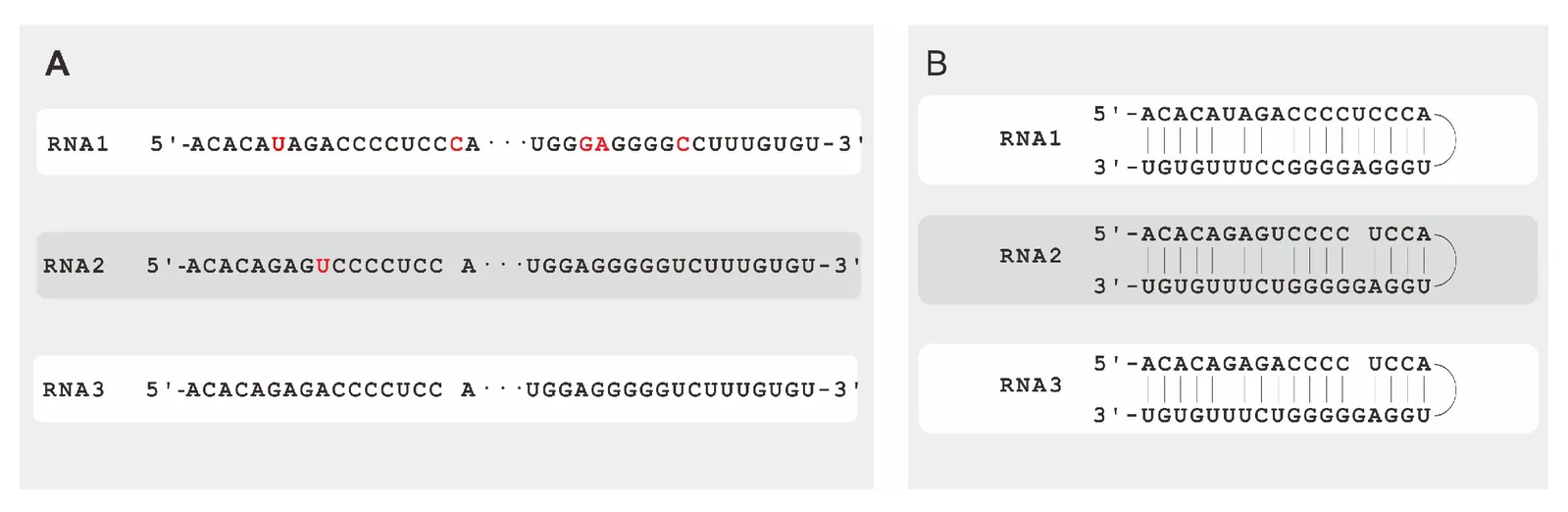
Terminal sequences of Bougainvillea progressive whitening virus (BgPWV). (**A**) Alignment of 5′ and 3′ termini of BgPWV RNAs. (**B**) Prediction of panhandle structures formed by the 5′ and 3′ termini of BgPWV RNAs.

Bioinformatic analysis of the putative open reading frames (ORFs) and conserved domains revealed that the two BgPWV isolates share the same genomic structure across their corresponding segments. Specifically, each of the three genomic RNAs from both isolates contains a single ORF flanked by untranslated regions (UTRs) (Figure 2, Table S2). For each segment, the putative proteins encoded by the two isolates were identical in length, had the same predicted molecular weights, and exhibited consistent conserved domain predictions. The amino acid sequence identities of the proteins encoded by RNA1, RNA2 and RNA3 between the two isolates were 98.04%, 99.21%, and 98.95%, respectively. The 5′ and 3′ terminal sequences of each RNA segment were highly conserved, and the two terminal regions of the same segment were complementary to each other (Figure 3), potentially forming a panhandle structure commonly found in other members of the order *Bunyavirales* [18,19,20].

The RNA1 had an AT content of approximately 70% and encoded a putative large protein (L) of 2599 aa (302 kDa). Bunyavirus RNA-dependent RNA polymerase (Bunya_RdRp) conserved domain (PFam04196, aa 789–1394) and OTU (Ovarian tumor) domain (cd22744, aa 2497–2598) were predicted in the L protein (Figure 2). No transmembrane helices, signal peptide, or N-glycosylation sites were predicted in the L protein. The 5′ and 3′ UTRs of RNA1 were 51 and 156 nt in length, respectively.

The RNA2 of BgPWV had an AT content of approximately 66% and encoded a putative viral movement protein (MP) of 378 aa (43 kDa). The viral MP conserved domain (PFam01107, aa 85–249) was predicted in the MP (Figure 2). A signal peptide with a cleavage site (VVS_24_NK) was identified using SignalP 4.1 (Figure S2a). Two N-glycosylation sites (N_236_LSR and N_350_STK) were predicted using NetNGlyc 1.0 (Figure S2b). The 5′ and 3′ UTRs of RNA2 are 58 and 127 nt, respectively.

RNA3 had an AT content of approximately 66% and encoded a putative nucleocapsid protein (N) of 287 aa (32 kDa). The Tenuivirus/Phlebovirus nucleocapsid protein (Tenui_N) conserved domain (PFam05733, aa 24–210) was predicted in the N protein (Figure 2). No transmembrane helices, signal peptide, or N-glycosylation sites were detected for the N protein. The difference in RNA3 length between the two isolates (1370 bp for a1 vs. 1361 bp for a2) was attributable solely to a 9-nt insertion (TGAATTCG) in the 5′ UTR of a1 at position 39 relative to a2. The coding regions were identical in length, and consequently both isolates encode the same 287-aa protein. The 5′ and 3′ UTRs of RNA3 are 68 or 60 and 438 or 437 nt, respectively. The 438 nt long 5′ UTR is highly rich in AT (~72%).

To further validate the assembled genomes and assess the completeness of each segment, clean reads were mapped back to the complete genome of BgPWV isolate a1 (used as the representative genome, since a1 and a2 share >98% sequence identity). A total of 18,975 reads mapped to RNA1, 4412 to RNA2, and 25,166 to RNA3, and the reads were distributed across the full length of each segment (Figure S3), supporting the completeness of the assembled genomes.

### 2.4. Sequence Comparisons, Phylogenetic Relationships and Recombination of BgPWV with Other Phenuivirids

BLASTn and BLASTx searches against the NCBI database showed that BgPWV a1 and a2 share the highest similarity with members of the genus *Rubodvirus* within the family *Phenuiviridae*. Subsequently, pairwise comparisons of BgPWV (a1 and a2) with members of different genera within the family *Phenuiviridae* were performed. The amino acid sequence identities of the L, MP, and N proteins between BgPWV (a1 and a2) and members of the genus *Rubodvirus* were 51.33–55.13%, 43.40–47.04%, and 57.65–61.57%, respectively, whereas the amino acid sequence identities between BgPWV and members of other genera within the family *Phenuiviridae* were below 21.55%, 14.55%, and 24.81%, respectively (Table 2, Figures S4–S6). In addition, nucleotide sequence identities between BgPWV and members of the genus *Rubodvirus* were 58.47–61.10%, 55.88–57.01%, and 58.09–60.51% for RNA1, RNA2, and RNA3, respectively (Table 2).

**Figure 4:**
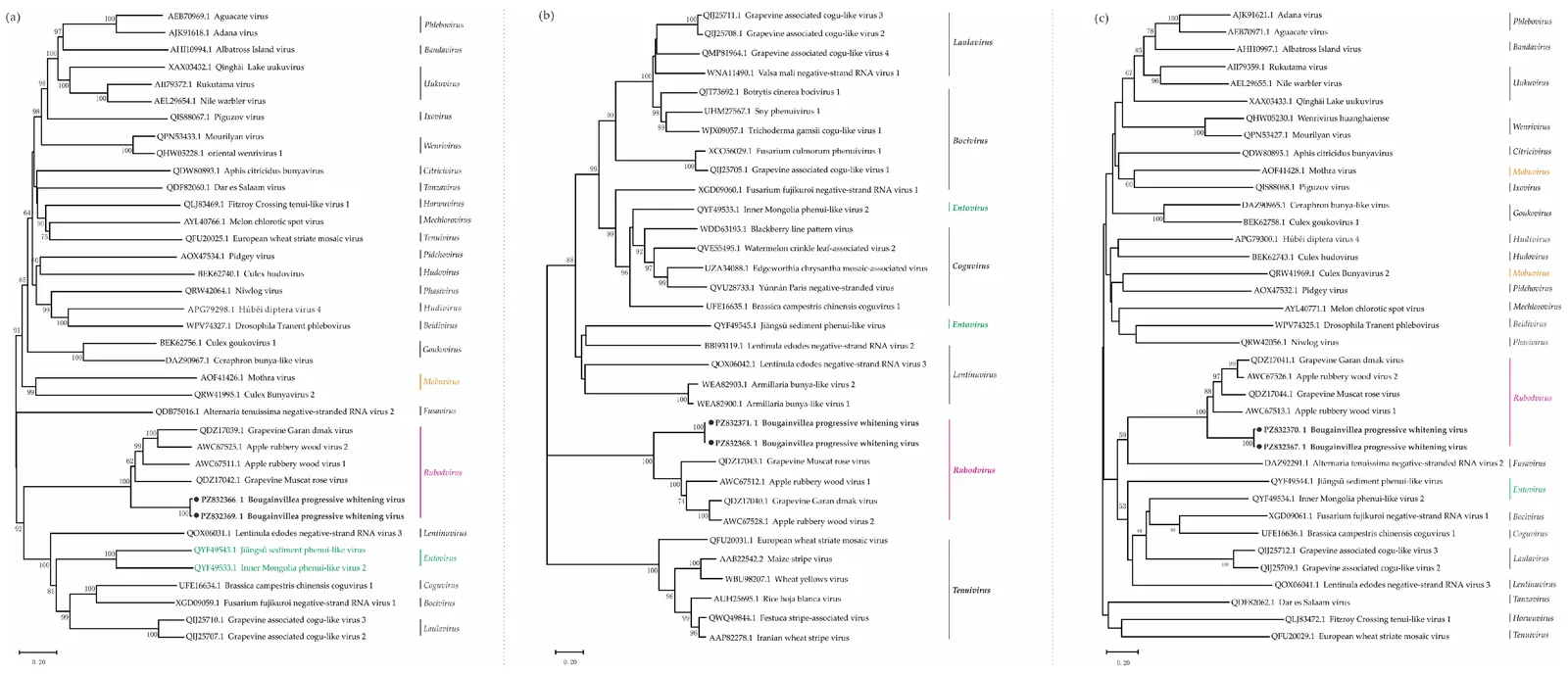
Neighbor-joining (NJ) phylogenetic trees constructed on the basis of amino acid sequences of the large protein (**a**), movement protein (**b**) and nucleocapsid protein (**c**) genes of BgPWV and the representative members of family *Phenuiviridae*. Black dots indicate the newly identified viruses in this study.

To clarify the taxonomic status of BgPWV, neighbor-joining (NJ) phylogenetic trees were constructed based on the amino acid sequences of the L, MP and N proteins. The results showed that the two isolates of BgPWV consistently clustered together in the phylogenetic trees of all three genes, forming a single monophyletic clade that was stably nested within the clade comprising known members of the genus *Rubodvirus* in the family *Phenuiviridae*. The bootstrap support values for the corresponding branches in the L, N, and MP trees were all 100%. This evolutionary lineage was distinctly separated from the representative viruses of the other 23 genera within the family, indicating a unique evolutionary position (Figure 4).

Notably, the phylogenetic topologies of some genera within the family were not entirely consistent across the different proteins: in the RdRp tree (Figure 4a), Mothra virus (AOF41428.1) and Culex Bunyavirus 2 (QRW41969.1), both belonging to the genus *Mobuvirus* clustered together on the same branch, whereas in the N tree (Figure 4c), they were resolved into two relatively independent branches. Similarly, in the RdRp tree, the two members of the genus *Entovirus* (Jiāngsū sediment phenui-like virus (JSPLV) and Inner Mongolia phenui-like virus 2 (IMPLV2)) initially clustered together and, along with the four genera *Lentinuvirus*, *Coguvirus*, *Bocivirus*, and *Laulavirus*, formed a large clade. However, although the MP and N trees still placed these five genera within a single large clade, their internal topologies were substantially rearranged: in the MP tree (Figure 4b), they were resolved into distinct subclades, with JSPLV clustering with lentinuvirus and IMPLV2 clustering with coguvirus, bocivirus, and laulavirus; in the N tree (Figure 4c), the entovirus members no longer preferentially grouped together.

No recombination events were detected between BgPWV a1 and a2, or between BgPWV (a1 and a2) and other phenuivirids.

### 2.5. Association Between the Viruses and the Symptoms

To further determine whether the creamy-white discoloration observed on bougainvillea leaves was associated with BgPWV infection, RT-PCR detection using BgPWV-specific primers (Table S1) was performed on leaf samples collected from all 14 bougainvillea plants located in the greenbelt described in Section 2.1. All 14 symptomatic samples tested positive for BgPWV. Since these symptoms were not observed elsewhere, 40 leaf samples were randomly collected from individual bougainvillea plants at other locations (Section 4.5), and all samples tested negative for BgPWV.

Given that three of the plants exhibiting creamy-white discoloration also exhibited chlorotic vein banding—a symptom similar to that caused by BCVBV—all collected samples were also tested using specific primers (see Section 4.5). Only the three samples exhibiting chlorotic vein banding symptoms tested positive, indicating that BCVBV and BgPWV can co-infect bougainvillea plants.

### 2.6. Inoculation Experiments

To preliminarily assess whether the newly discovered virus (BgPWV) could be mechanically transmitted from symptomatic bougainvillea plants to asymptomatic bougainvillea and *N. benthamiana* plants, mechanical inoculation experiments were performed. All inoculated bougainvillea plants and *N. benthamiana* plants remained asymptomatic throughout the observation period. Further RT-PCR testing at 30 and 60 days post-inoculation (dpi) confirmed the absence of BgPWV in all inoculated plants.

### 2.7. Preliminary Investigation of Potential Transmission Vectors

To preliminarily explore the potential vectors involved in the field transmission of BgPWV, a preliminary survey of invertebrates associated with bougainvillea plants was conducted in the greenbelt described in Section 2.1. No obvious pest infestations or large populations of invertebrates were observed during the collection periods. Among the examined plants, only the giant African snail (*Achatina fulica*) was sporadically found on the leaves. RT-PCR detection of BgPWV in the collected snails yielded negative results, suggesting that this species is unlikely to serve as a vector for BgPWV, at least under the field conditions examined in this study.

## 3. Discussion

Bougainvillea is an important ornamental plant, particularly in tropical and subtropical regions. In this study, a novel virus, provisionally named Bougainvillea progressive whitening virus (BgPWV), was identified in bougainvillea plants exhibiting progressive leaf whitening in Haikou, Hainan Province, China. BgPWV has a tripartite, negative-sense RNA genome encoding the L, MP, and N proteins, a genomic organization typical of the genus *Rubodvirus* (family *Phenuiviridae*) [21,22,23]. The highly conserved and complementary terminal sequences of each segment are predicted to form panhandle structures, a feature common to members of the order *Bunyavirales* and thought to be important for genome replication and packaging [20].

Phylogenetic analyses based on the L, MP, and N proteins consistently placed BgPWV within the genus *Rubodvirus*, forming a monophyletic clade with strong bootstrap support (100%). Pairwise comparisons based on L protein amino acid sequences showed that the two BgPWV isolates (a1 and a2) shared the highest identity with apple rubbery wood virus 1 (ARWV1), at 55.13% and 54.88%, respectively (Table 2). These values are well below the 95% species demarcation threshold of the genus *Rubodvirus* [24], indicating that BgPWV represents a relatively divergent new species. Accordingly, the name *Rubodvirus haikouense* is proposed for this new species. Moreover, the low sequence identity between BgPWV and its closest relatives suggests that additional, as-yet-unidentified rubodviruses may still be discovered through broader sampling of plant hosts. Finally, the phylogenetic incongruence among the L, MP, and N trees for some genera (e.g., *Mobuvirus*, *Entovirus*) (Figure 4) highlights the value of using multiple genomic segments for taxonomic inference in this family, since single-gene trees may not fully resolve evolutionary relationships.

The genus *Rubodvirus* currently comprises four formally classified viruses—apple rubbery wood virus 1 and 2, grapevine Muscat rose virus, and grapevine Garan dmak virus—whose genomes typically consist of three negative-sense RNA segments encoding the L, MP, and N proteins, although additional RNA segments have been reported in some isolates of apple rubbery wood virus 2. *Rubodvirus* species were first identified in apple [21] and have subsequently been reported in other woody hosts, including grapevine [22], pear [25,26], raspberry [27] and quince [28]. The discovery of BgPWV not only adds a new member to this genus but also extends its known host range to the genus *Bougainvillea*, highlighting the potential for rubodviruses to infect diverse ornamental plants.

A strong association was observed between BgPWV infection and the creamy-white leaf symptoms: all 14 symptomatic plants tested positive for BgPWV, whereas all 40 samples collected from other locations where the symptoms were absent tested negative. However, Koch’s postulates were not fulfilled in this study, and a causal relationship between BgPWV infection and the progressive leaf whitening phenotype therefore remains to be established. Thus, the possibility that BgPWV is not the sole causal agent, or that additional factors (e.g., co-infecting viruses, host genotype, or environmental conditions) contribute to symptom development, cannot be excluded.

Notably, three of the 14 symptomatic plants were also co-infected with BCVBV, a badnavirus (family *Caulimoviridae*) that has previously been associated with chlorotic vein-banding in bougainvillea [3,4,5,6,7,8,9,10,11]. The chlorotic vein-banding observed on these three plants was consistent with BCVBV infection, whereas the remaining 11 plants, which were positive only for BgPWV, exhibited progressive creamy-white leaf discoloration without vein-banding. This pattern suggests that BgPWV and BCVBV may induce distinct symptoms in bougainvillea, and that the two viruses can co-exist in the same host. Mixed infections involving DNA and RNA viruses are not uncommon in perennial woody and ornamental hosts [29,30,31], where co-infecting viruses may interact synergistically, antagonistically, or independently [32,33,34]. In the present study, no obvious difference in symptom severity was observed between the three co-infected plants and the 11 plants infected with BgPWV alone; however, the small number of co-infected plants precludes any firm conclusion regarding virus–virus interactions.

To date, BgPWV has only been detected in Haikou, Hainan, and systematic information regarding its geographical distribution is currently lacking. Mechanical inoculation of *N. benthamiana* and bougainvillea seedlings failed to transmit the virus (Section 2.6), and a preliminary survey of potential vectors on bougainvillea plants found no insects; Only the giant African snail was observed, and it tested negative for BgPWV (Section 2.7). Given the limited scope of this survey, the possibility of other potential vectors—such as whiteflies, aphids, or thrips—cannot be excluded. In addition to vector transmission, seed transmission represents another important route for the spread of many negative-sense RNA viruses, including members of the family *Phenuiviridae* [35,36]. In the present study, however, no flowering or seed production was observed in the surveyed bougainvillea plants, and therefore seed transmission of BgPWV could not be experimentally evaluated. Future work using seeds from infected plants, once available, will be necessary to determine whether BgPWV can be transmitted vertically through seeds and to assess its potential role in the long-distance spread of the virus. It should be noted that the present study focused primarily on the identification and molecular characterization of BgPWV; the biological experiments were limited to PCR-based virus detection, and the transmission mechanism remains unresolved. Further studies are therefore needed to identify the transmission vector(s) of BgPWV, to assess its potential for seed transmission, and to elucidate its epidemiological dynamics under field conditions.

In conclusion, we report the identification and genomic characterization of a novel rubodvirus, BgPWV, infecting bougainvillea in China. Our findings expand the known diversity of the genus *Rubodvirus* and provide a foundation for future studies on the biology, epidemiology, and management of viral diseases in bougainvillea. Further research is needed to determine the full host range, transmission mechanism, and economic impact of BgPWV, as well as to develop effective management strategies to mitigate its spread in ornamental production systems.

## 4. Materials and Methods

### 4.1. Plant Materials, RNA Extraction, High-Throughput Sequencing (HTS), and Virus Annotation

Two bougainvillea plants (designated a1 and a2) showing creamy-white leaf margins were initially sampled from Haikou City, Hainan Province, China. Total RNA was extracted from leaf tissues of each plant using TRIzol Reagent (Invitrogen, Waltham, MA, USA). Equal amounts of RNA from samples a1 and a2 were pooled to form a single mixed sample, designated PL-1, for high-throughput sequencing library construction. Ribosomal RNA (rRNA) depletion was performed using the Ribo-Zero Gold rRNA Removal Kit (Illumina, San Diego, CA, USA), and subsequent library preparation was carried out at Shanghai Majorbio Bio-Pharm Biotechnology Co., Ltd. (Shanghai, China). The library was sequenced using a 2 × 150 bp paired-end configuration on an Illumina NovaSeq 6000 platform according to the manufacturer’s instructions.

After adapter trimming and quality filtering of the raw reads, clean reads were aligned to the *Bougainvillea glabra* reference genome (GCA_045838725.1) using HISAT2 v2.1.0 [37]; the mapped reads were removed to reduce dataset complexity, and the remaining unmapped reads were assembled de novo into contigs using SPAdes [38]. The resulting contigs were annotated using BLASTn and BLASTx [39] against the NCBI non-redundant nucleotide (nt) and protein (nr) databases [40], using conservative e-value cut-offs of 1 × 10^−4^ and 1 × 10^−6^, respectively. The completed genomes of BgPWV (isolate a1) were used as the reference sequences for read mapping using bowtie2 [41].

### 4.2. RT-PCR Assay and Genome Reconstruction of the Novel Virus

Based on the viral contigs assembled from the high-throughput sequencing data, specific primers (Table S1) were designed to validate the presence of the novel virus in the original samples. The RNA samples extracted from a1 and a2 in Section 4.1 were reverse-transcribed into cDNA using the TransScript One-Step gDNA Removal and cDNA Synthesis SuperMix (TransGen, Beijing, China). RT-PCR was performed using the resulting cDNA as the template to determine whether both samples were positive for the novel virus, and only the positive samples were used for subsequent genome reconstruction.

To obtain the complete sequences of the viral RNAs, each viral RNA segment was amplified to cover nearly its full length. For RNA1, four primer pairs (Table S3) were designed to generate four overlapping amplicons (>2.0 kb each), which together covered almost the entire RNA1. For RNA2 and RNA3, one primer pair (Table S3) was used for each segment to generate a single amplicon of approximately 1.2 kb, covering nearly the full length of each segment. The 5′ and 3′ terminal sequences were determined using a SMARTer^®^ RACE 5′/3′ Kit (Takara, San Jose, CA, USA). Since the viral genome naturally lacks a poly(A) tail, the first-strand cDNA synthesis for 5′-RACE was performed following the random-priming protocol provided in the kit, which simultaneously introduces the universal primer-binding site. For 3′-RACE, a poly(A) tail was first added to the 3′ terminus of the RNA using Poly(A) Polymerase (Takara, San Jose, CA, USA), and the first-strand cDNA was then synthesized using the 3′-RACE CDS Primer A (provided in the kit), thereby incorporating the universal primer-binding site. Subsequently, touchdown PCR was performed using the Universal Primer A Mix (UPM) and gene-specific primers (GSPs; Table S3) designed from the viral contigs, with the 5′- and 3′-RACE-ready cDNAs as templates, to amplify the terminal sequences.

The amplified PCR products were purified using a TIANgel Midi Purification Kit (TianGen, Beijing, China). The purified products were either directly sequenced by Sanger sequencing or cloned into the pMD18-T vector (Takara, San Jose, CA, USA). The recombinant vectors were subsequently transformed into *Escherichia coli* DH5α competent cells (Takara, Japan). Three positive clones were selected for each PCR product and sequenced bidirectionally by Sanger sequencing at Sangon Biotech (Sanya, China).

### 4.3. Sequence and Phylogenetic Analyses

Open reading frames (ORFs) were predicted using the ORFfinder online tool (https://www.ncbi.nlm.nih.gov/orffinder/; accessed on 3 July 2026), and conserved domains and motifs were identified by searching the Conserved Domain Database (CDD, https://www.ncbi.nlm.nih.gov/Structure/cdd/wrpsb.cgi; accessed on 3 July 2026). Molecular weights of encoded proteins were predicted by an online tool of Expasy (https://web.expasy.org/compute_pi/; accessed on 3 July 2026). Transmembrane domains and signal sequences were predicted with SignalP 4.1 and TMHMM programs on the DeepTMHMM [42].

Pairwise comparisons were performed based on CLUSTALW multiple sequence alignments, and NJ phylogenetic trees were constructed using MEGA 11 with 1000 bootstrap iterations [43].

The aa sequences of member species (RdRP, GP, and NP of emaravirus; RdRP, CP, and HSP70h of crinivirus) were obtained from the International Committee on Taxonomy of Viruses (ICTV) report and NCBI database.

### 4.4. Recombination Analysis

Recombination events were detected using the Recombination Detection Program (RDP) version v5.29 with default parameter values [44]. The genomic sequences used for recombination analysis included BgPWV-a1, BgPWV-a2, and representative sequences of all species in the family *Phenuiviridae*. The sequences were aligned in MEGA 11 software. Subsequently, the aligned nucleotide sequences were imported into RDP and analyzed using seven detection algorithms (RDP, GENECONV, BootScan, Chimaera, MaxChi, SiScan, and 3Seq). The potential recombination events were considered significant only when supported by at least four different algorithms with *p*-value < 10^−6^ and combination scores above 0.6.

### 4.5. Screening of the Viruses

To investigate the viral etiology associated with the creamy-white leaf margin symptoms, leaf samples were collected from individual bougainvillea plants at multiple locations in Hainan. Specifically, all 14 plants located in a single greenbelt in Haikou (described in Section 2.1) were sampled individually. In addition, 40 asymptomatic leaf samples were randomly collected from bougainvillea plants in other greenbelts across Hainan, including Haikou (10), Sanya (10), Lingshui (6), Wenchang (5), and Qionghai (9), where no such symptoms had been observed.

For BgPWV detection, total RNA was extracted from each of the 54 leaf samples using the RNAPrep Pure Plant Kit (TianGen, Beijing, China). First-strand cDNA was synthesized from the extracted RNA using random hexamers and the TransScript One-Step gDNA Removal and cDNA Synthesis SuperMix (TransGen Biotech, Beijing, China) according to the manufacturer’s protocol. RT-PCR was subsequently performed using three pairs of BgPWV-specific primers targeting RNA1, RNA2, and RNA3, respectively: BgPWV-RNA1-F/R, BgPWV-RNA2-F/R, and BgPWV-RNA3-F/R (Table S1).

For BCVBV detection, given that three of the symptomatic plants also exhibited chlorotic vein banding—a symptom resembling BCVBV infection—total DNA was extracted from all 54 leaf samples using the Hi-DNAsecure Plant Kit (TianGen, Beijing, China). PCR was then performed using the BCVBV-specific primers BadnaF (5′-AAAGACCATGAGAAGCATC-3′) and BadnaR (5′-ATAAGCATCAGGGGGTG-3′) [6].

### 4.6. Mechanical Inoculation

Leaves of bougainvillea plants exhibiting creamy-white leaf symptoms and containing the putative novel virus were homogenized with a mortar and pestle in 0.02 M sodium phosphate buffer (pH 7.4) to obtain crude sap. The sap extract was mixed with silicon carbide and gently rubbed onto the leaves of ten *N. benthamiana* seedlings (each possessing 3–4 fully expanded leaves) and bougainvillea seedlings (approximately 20 cm in height). All the *N. benthamiana* plants and bougainvillea seedlings were maintained in the insect-proof net house. RT-PCR assays were performed to detect BgPWV at 30 and 60 days post-inoculation (dpi) using three pairs of BgPWV-specific primers targeting RNA1, RNA2, and RNA3, respectively: BgPWV-RNA1-F/R, BgPWV-RNA2-F/R, and BgPWV-RNA3-F/R (Table S1).

### 4.7. Survey and Detection of Potential Transmission Vectors

To investigate potential vectors involved in the field spread of BgPWV, all visible invertebrates were collected from the above-ground parts (leaves, stems, and branches) of bougainvillea plants at the sampling sites described in Section 2.1 using forceps and aspirators, and the collected organisms were identified based on their morphological characteristics. Total RNA was extracted from the collected invertebrates using the FastPure Composite Tissue/Cell Total RNA Extraction Kit (Vazyme, Nanjing, China), and first-strand cDNA was synthesized using the TransScript One-Step gDNA Removal and cDNA Synthesis SuperMix (TransGen, Beijing, China) according to the manufacturer’s instructions. BgPWV was detected by RT-PCR using the same BgPWV-specific primers and operational procedures as described in Section 4.6.

## 5. Conclusions

In this study, we identified and characterized a novel tripartite negative-sense RNA virus, Bougainvillea progressive whitening virus (BgPWV), infecting bougainvillea plants exhibiting progressive leaf whitening in Hainan Province, China. Based on its genome organization, sequence identities, and phylogenetic placement, BgPWV is a member of a new species of the genus *Rubodvirus* (family *Phenuiviridae*), thereby extending the known host range of rubodviruses to the genus *Bougainvillea*. A strong association between BgPWV infection and the creamy-white leaf phenotype was supported by RT-PCR detection of field samples, although Koch’s postulates were not fulfilled and a causal relationship remains to be confirmed. Co-infection with BCVBV in three plants indicates that mixed viral infections occur in bougainvillea and may complicate symptom-based diagnosis. The transmission routes of BgPWV remain unknown, and further studies are needed to identify its vector(s), assess whether it can be seed-transmitted, determine its full host range, and evaluate its economic impact on ornamental production. While the biological characterization of BgPWV is still limited, the genomic and phylogenetic data presented here provide a solid basis for subsequent studies on its transmission, host range, and epidemiology. Overall, this study expands the known viral diversity associated with bougainvillea and offers valuable information for the management of viral diseases in this ornamental plant.

## Figures and Tables

**Table 1:** BLASTx annotation of contigs assembled from the bougainvillea mixed sample PL-1.

Taxonomy	Blastx_Hit	No. of Contigs	Length Range (nt)	Alignment Length (nt)	Identity Range (%)
*Phenuiviridae*, *Rubodvirus*	Apple rubbery wood virus 1	7	234–7983	231–7074	54–74.8
	Apple rubbery wood virus 2	2	222–1385	222–885	48.6–53.1
	Grapevine Garan dmak virus	1	1383	858	54.4

**Table 2:** Pairwise identities (%) of nucleotide (nt) and amino acid (aa) sequences between Bougainvillea progressive whitening virus (BgPWV) and other members of genus *Rubodvirus*.

Virus Name(s)	Species	GenBank Accessions	Segments	Percent nt Identity *	Percent aa Identities
Grapevine Muscat rose virus	*Rubodvirus argentinaense*	MK728654	RNA1	58.65/58.47	51.52/51.33 (L)
		MK728655	RNA2	56.28/56.44	43.48/43.75 (MP)
		MK728656	RNA3	58.96/59.42	58.01/57.65 (N)
Grapevine Garan dmak virus	*Rubodvirus armeniaense*	MK728657	RNA1	59.66/59.6	52.45/52.49 (L)
		MK728658	RNA2	55.88/56.04	47.04/47.04 (MP)
		MK728659	RNA3	60.29/60.51	59.57/58.87 (N)
Apple rubbery wood virus 1	*Rubodvirus mali*	MF062125	RNA1	60.73/60.77	55.13/54.88 (L)
		MF062126	RNA2	56.44/56.52	43.4/43.4 (MP)
		MF062127	RNA3	58.37/58.98	61.57/61.57 (N)
Apple rubbery wood virus 2	*Rubodvirus prosserense*	MF062139	RNA1	61.1/60.93	54.7/54.53 (L)
		MF062142	RNA2a	56.7/57.01	46.63/46.9 (MP)
		MF062143	RNA2b	55.99/56.15	43.7/43.97 (MP)
		MF062140	RNA3a	60.18/60.48	59.93/59.22 (N)
		MF062141	RNA3b	58.09/58.14	60.14/60.14 (N)

* Values before and after the slash represent the sequence identities of BgPWV isolates a1 and a2, respectively, with the corresponding segments of the reference viruses.

## Data Availability

The complete genome sequences of two BgPWV isolates have been deposited in GenBank under accession numbers PZ832366–PZ832371. Specifically, for isolate a1, RNA1, RNA2, and RNA3 correspond to PZ832366, PZ832368, and PZ832367, respectively; for isolate a2, RNA1, RNA2, and RNA3 correspond to PZ832369, PZ832371, and PZ832370, respectively.

## Supplementary Materials

The following supporting information can be downloaded at: https://www.mdpi.com/article/10.3390/plants15203193/s1. Figure S1. RT-PCR detection of the three genomic segments (RNA1–3) of Bougainvillea progressive whitening virus (BgPWV) in samples a1 and a2. DL 2000 DNA Marker; a1, a2: samples tested; NC, negative control.; Figure S2. Signal peptide (a) and N-glycosylation sites (b)predicted in movement protein (MP) of Bougainvillea progressive whitening virus (BgPWV); Figure S3. Distribution of reads mapped to the complete genome of Bougainvillea progressive whitening virus (BgPWV); Figure S4. Pairwise amino acid sequence identities (%) of the L proteins between BgPWV (a1 and a2) and representative members of the family *Phenuiviridae*; Figure S5. Pairwise amino acid sequence identities (%) of the MP proteins between BgPWV (a1 and a2) and representative members of the family *Phenuiviridae*; Figure S6. Pairwise amino acid sequence identities (%) of the N proteins between BgPWV (a1 and a2) and representative members of the family *Phenuiviridae*; Table S1. Primers for amplifying prevalence investigation of Bougainvillea progressive whitening virus (BgPWV); Table S2. Genome organization and ORF/UTR positions of Bougainvillea progressive whitening virus (BgPWV); Table S3. Primers used to complete the genomes of Bougainvillea progressive whitening virus (BgPWV).
