# Supplementary material for: A Novel Rubodvirus Associated with Progressive Leaf Whitening Disease in Bougainvillea: Genomic Insights and Phylogenetic Relationships
Source: Plants (Basel). 2026 Oct 18;15(20):3193. doi: 10.3390/plants15203193 (PMC13709196; doi:10.3390/plants15203193)
Supplement: Supplementary file 1 [file plants-15-03193-s001.zip › plants-4607956-supplementary.pdf]

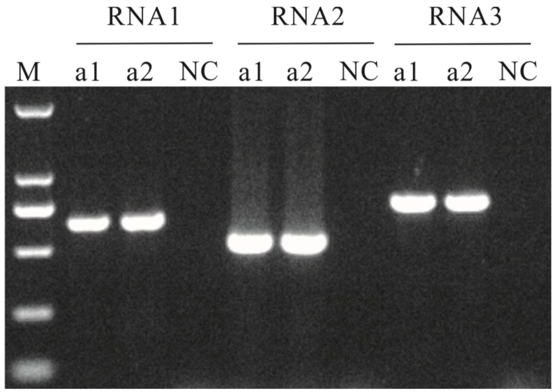

Figure S1. RT-PCR detection of the three genomic segments (RNA1–3) of Bougainvillea progressive whitening virus (BgPWV) in samples a1 and a2. DL 2 000 DNA Marker; a1, a2: samples tested; NC, negative control.

(a)

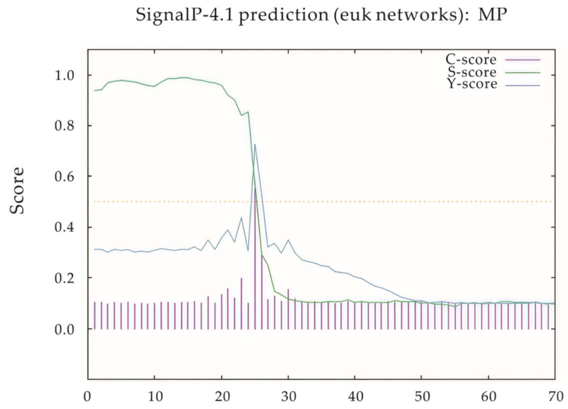

(b)

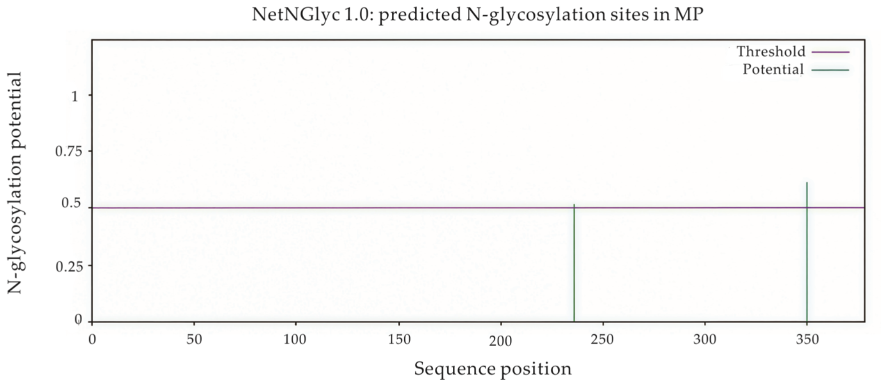

Figure S2. Signal peptide (a) and N-glycosylation sites (b)predicted in movement protein (MP) of Bougainvillea progressive whitening virus (BgPWV)

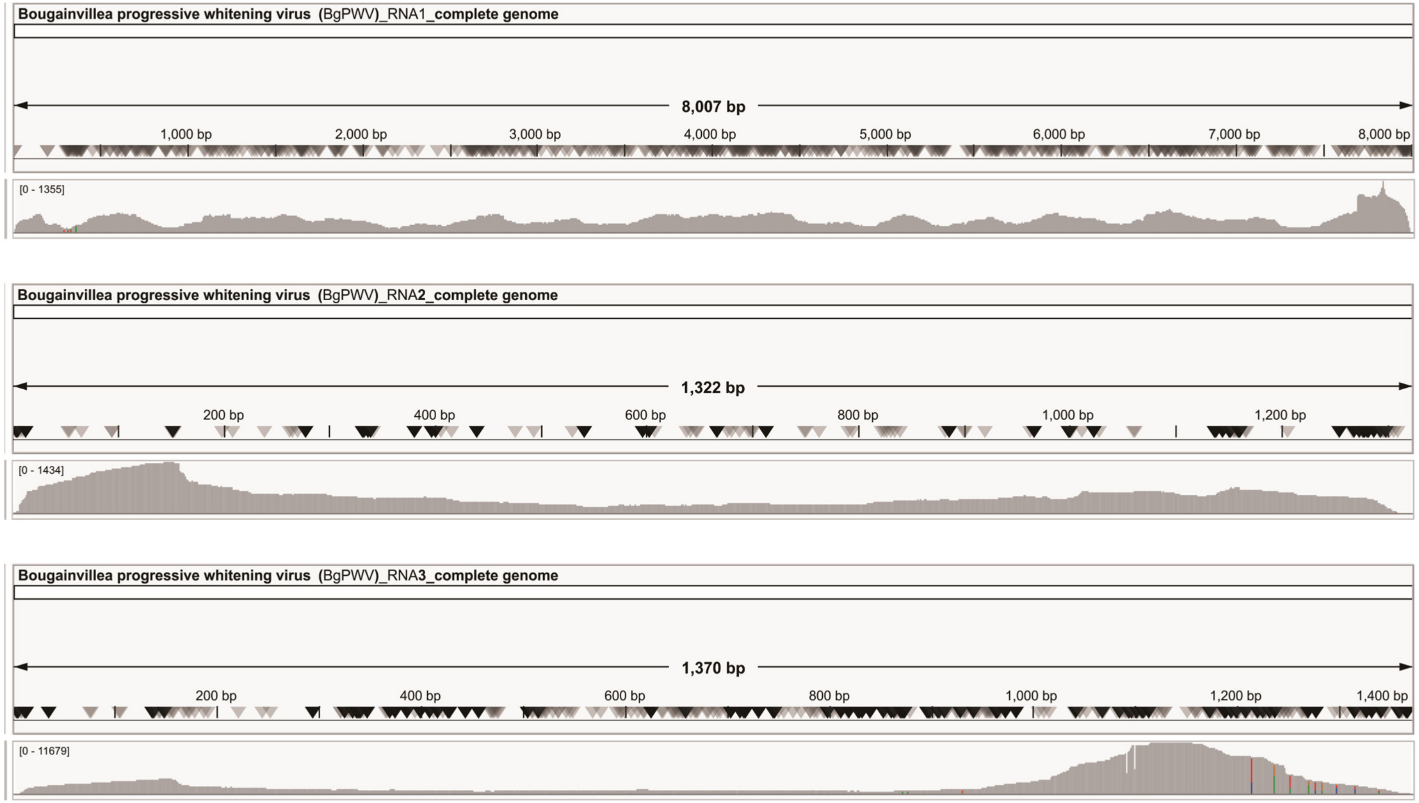

Figure S3. Distribution of reads mapped to the complete genome of Bougainvillea progressive whitening virus (BgPWV)

Virus species	Accession	No.	1	2	3	4	5	6	7	8	9	10	11	12	13	14	15	16	17	18	19	20	21	22	23	24	25	26	27	28	29	30	31	32	33	34	35	36	37
Rubodvirus haikouense (isolate a1)	This study	1	100	98.04	51.52	52.45	54.7	55.13	19.32	19.25	19.54	19.21	20.3	19.99	17.99	19.51	19.78	19.72	18.12	19.68	19.27	18.87	19.97	20.07	19.63	20.42	19.16	19.71	19.28	21.55	17.18	18.14	20.4	20.79	19.45	19.44	19.13	18.26	19.51
Rubodvirus haikouense (isolate a2)	This study	2	98.04	100	51.33	52.49	54.53	54.88	19.52	19.45	19.59	19.05	20.25	20.15	18.04	19.56	19.72	19.78	18.07	19.68	19.17	19.02	19.83	20.16	19.58	20.58	19.16	19.71	19.43	21.55	17.23	18.14	20.55	20.84	19.64	19.53	19.27	18.39	19.69
Rubodvirus argentinaense	QDZ17042.1	3	51.52	51.33	100	53.92	56.48	55.11	20.3	20.5	20.84	19.66	20.18	20.85	19.56	20.18	20.35	20.35	19.5	20.67	19.74	18.62	20.14	20.6	19.85	20.18	19.6	20.43	19.71	21.13	17.39	18	21.14	20.26	18.82	18.58	20.37	19.05	19.44
Rubodvirus armeniaense	QDZ17039.1	4	52.45	52.49	53.92	100	67.38	58.33	19.13	18.61	19.81	18.79	19.47	20.51	18.08	19.63	19.26	19.63	17.93	18.87	18.36	18.19	19.74	19.47	18.53	18.72	18.66	19.3	18.87	20.32	17.14	17.71	19.77	19.38	18.3	18.16	18.71	17.82	18.13
Rubodvirus prosserense	AWC67525.1	5	54.7	54.53	56.48	67.38	100	60.4	20.19	19.1	20.68	18.95	19.64	19.75	18.22	19.4	19.65	19.93	18.24	19.48	18.21	17.34	20.4	20.02	18.89	19.46	19.41	19.94	18.79	20.91	17.46	18.03	20.06	19.68	19.78	19.05	18.43	19.15	19.15
Rubodvirus mali	AWC67511.1	6	55.13	54.88	55.11	58.33	60.4	100	21.1	19.75	21.01	20.38	20.28	20.32	18.91	20.11	20.61	20.24	19.78	20.42	19.13	18.54	20.79	20.31	19.81	19.57	20.21	20.09	19.11	22.41	18.28	17.98	20.36	20.18	18.93	19.18	19.8	20	19.49
Hudivirus muscae	APG79298.1	7	19.32	19.52	20.3	19.13	20.19	21.1	100	32.96	26.36	24.84	27.67	27.11	25.39	25.31	25.75	26.2	24.85	24.48	23.21	23.47	25.26	25.6	24.29	24.32	23.46	27.59	24.32	22.79	21.06	20.63	21.01	20.32	20.39	19.86	20.38	19.07	19.02
Beidivirus drosophilae	WPV74327.1	8	19.25	19.45	20.5	18.61	19.1	19.75	32.96	100	25.39	24.86	27.93	26.88	24.93	26.16	25.68	26.46	24.42	24.9	23.52	25.21	26.52	26.15	26.9	23.5	22.15	27.9	24.11	22.45	21.24	19.42	19.5	19.89	20.02	19.43	19.52	17.76	19
Phlebovirus aguacateense	AEB70969.1	9	19.54	19.59	20.84	19.81	20.68	21.01	26.36	25.39	100	60.57	37.93	38.19	33.56	34.12	29.99	30.57	30.57	29.09	26.94	24.86	27.94	26.03	26.83	26.93	24.64	25.36	24.5	23.76	21.42	22.22	21.66	21.99	20.36	20.62	21.08	20.59	20.13
Phlebovirus adanaense	AJK91618.1	10	19.21	19.05	19.66	18.79	18.95	20.38	24.84	24.86	60.57	100	37.3	36.62	31.78	34.33	29.73	30.15	29.6	29.25	27.2	25.09	27.8	27.18	26.82	27.52	25.32	24.78	24.99	23.79	22.22	22.61	21.12	20.3	19.92	20.04	22.04	20.57	19.97
Uukuvirus rukutamaense	AII79372.1	11	20.3	20.25	20.18	19.47	19.64	20.28	27.67	27.93	37.93	37.3	100	62.78	39.2	35.48	30.38	31.92	32.27	31.1	28.59	26.59	28.3	27.8	28.86	26.56	26.41	25.73	25.12	25.36	20.78	22.31	22.53	22.33	21.22	21.56	21.65	20.93	20.29
Uukuvirus schmidti	AEL29654.1	12	19.99	20.15	20.85	20.51	19.75	20.32	27.11	26.88	38.19	36.62	62.78	100	39.66	35.48	32.63	32.54	32.89	31	28.19	27.22	27.18	27.97	28.1	27.22	26.2	26.44	26.11	25.57	21.36	23.23	22.25	22.28	22.41	21.96	21.73	20.96	20.57
Uukuvirus qinghaiense	XAX03432.1	13	17.99	18.04	19.56	18.08	18.22	18.91	25.39	24.93	33.56	31.78	39.2	39.66	100	30.84	29.7	30.66	29.17	27.97	27.09	26.34	27.08	26.52	27.2	25.82	25.14	24.88	24.48	23.99	20.86	22.14	20.25	19.13	19.86	19.49	19.88	18.72	18.65
Bandavirus albatrossense	AHI10994.1	14	19.51	19.56	20.18	19.63	19.4	20.11	25.31	26.16	34.12	34.33	35.48	35.48	30.84	100	28.48	28.85	28.81	26.99	25.46	24.53	25.88	26.64	25.23	26.67	24.77	23.8	22.96	24.04	22.26	22.61	21.16	20.33	20.48	20.16	20.78	20.48	19.47
Wenrivirus penaei	QPN53433.1	15	19.78	19.72	20.35	19.26	19.65	20.61	25.75	25.68	29.99	29.73	30.38	32.63	29.7	28.48	100	79.51	31.96	29.11	28.72	26.54	29.34	28.14	27.43	28.02	27.27	26.33	23.87	26.67	22.85	22.62	21.36	22.16	21.09	20.78	20.24	19.95	19.59
Wenrivirus huanghaiense	QHW05228.1	16	19.72	19.78	20.35	19.63	19.93	20.24	26.2	26.46	30.57	30.15	31.92	32.54	30.66	28.85	79.51	100	31.5	28.86	28.46	26.93	29.69	27.98	27.93	29.05	27.43	26.87	24.52	26.29	22.75	22.3	21.79	22	21.14	20.99	20.49	19.78	19.63
Ixovirus antarcticaense	QIS88067.1	17	18.12	18.07	19.5	17.93	18.24	19.78	24.85	24.42	30.57	29.6	32.27	32.89	29.17	28.81	31.96	31.5	100	27.31	26.57	25.43	26.63	26.95	25.67	25.89	24.64	24.45	23.54	24.11	20.96	21.51	21.41	20.96	20.41	20.23	20.95	19.17	18.98
Citricivirus chongqinense	QDW80893.1	18	19.68	19.68	20.67	18.87	19.48	20.42	24.48	24.9	29.09	29.25	31.1	31	27.97	26.99	29.11	28.86	27.31	100	27.74	25.76	26.5	27.26	29.08	27.77	25.78	25.11	25.14	24.71	21.17	20.7	21.4	21.86	20.57	20.47	20.63	18.67	18.74
Tanzavirus daressalaamense	QDF82060.1	19	19.27	19.17	19.74	18.36	18.21	19.13	23.21	23.52	26.94	27.2	28.59	28.19	27.09	25.46	28.72	28.46	26.57	27.74	100	25.58	26.37	25.9	27.59	25.87	26.12	23.38	23.89	22.89	20.61	22.16	20.04	19.86	20.34	20.07	20.01	18.46	19.37
Pidchovirus pidgei	AOX47534.1	20	18.87	19.02	18.62	18.19	17.34	18.54	23.47	25.21	24.86	25.09	26.59	27.22	26.34	24.53	26.54	26.93	25.43	25.76	25.58	100	25.38	26.32	26.45	25.1	24.2	23.01	24.69	22.07	20.23	20.05	19.97	19.74	20.4	20.52	19.29	18.75	17.67
Mechlorovirus cucumeris	AYL40766.1	21	19.97	19.83	20.14	19.74	20.4	20.79	25.26	26.52	27.94	27.8	28.3	27.18	27.08	25.88	29.34	29.69	26.63	26.5	26.37	25.38	100	31.37	29.66	26.03	24.9	26.18	23.57	23.9	21.36	20.51	21.69	21.93	19.6	18.98	21.24	19.22	18.87
Tenuivirus eurotritici	QFU20025.1	22	20.07	20.16	20.6	19.47	20.02	20.31	25.6	26.15	26.03	27.18	27.8	27.97	26.52	26.64	28.14	27.98	26.95	27.26	25.9	26.32	31.37	100	29.64	26.22	24.77	25.75	24.01	24.25	20.97	20.64	21.41	20.88	19.45	19.26	19.93	19.05	18.63
Horwuvirus fitzroyense	QLJ83469.1	23	19.63	19.58	19.85	18.53	18.89	19.81	24.29	26.9	26.83	26.82	28.86	28.1	27.2	25.23	27.43	27.93	25.67	29.08	27.59	26.45	29.66	29.64	100	26.34	24.05	25.7	23.12	24.05	21.44	20.84	21.1	21.9	18.88	18.27	20.3	18.58	18.6
Goukovirus culicis	BEK62756.1	24	20.42	20.58	20.18	18.72	19.46	19.57	24.32	23.5	26.93	27.52	26.56	27.22	25.82	26.67	28.02	29.05	25.89	27.77	25.87	25.1	26.03	26.22	26.34	100	44.34	23.95	22.88	26.82	23.29	22.11	20.64	21.34	19.64	19.12	20.98	20.58	19.99
Goukovirus ceraphri	DAZ90967.1	25	19.16	19.16	19.6	18.66	19.41	20.21	23.46	22.15	24.64	25.32	26.41	26.2	25.14	24.77	27.27	27.43	24.64	25.78	26.12	24.2	24.9	24.77	24.05	44.34	100	22.83	22.48	24.5	21.15	21.58	20.11	20.2	18.94	18.84	19.02	18.67	18.85
Phasivirus alamedaense	QRW42064.1	26	19.71	19.71	20.43	19.3	19.94	20.09	27.59	27.9	25.36	24.78	25.73	26.44	24.88	23.8	26.33	26.87	24.45	25.11	23.38	23.01	26.18	25.75	25.7	23.95	22.83	100	22.11	22.59	21.53	20.69	20.52	21.03	19.29	19.91	19.61	18.97	18.19
Hudovirus culicis	BEK62740.1	27	19.28	19.43	19.71	18.87	18.79	19.11	24.32	24.11	24.5	24.99	25.12	26.11	24.48	22.96	23.87	24.52	23.54	25.14	23.89	24.69	23.57	24.01	23.12	22.88	22.48	22.11	100	21.42	20.92	18.25	19.25	19.36	20.11	18.93	19.71	18.76	18.23
Fusavirus alternariae	QDB75016.1	28	21.55	21.55	21.13	20.32	20.91	22.41	22.79	22.45	23.76	23.79	25.36	25.57	23.99	24.04	26.67	26.29	24.11	24.71	22.89	22.07	23.9	24.25	24.05	26.82	24.5	22.59	21.42	100	20.84	21.06	23.55	23.23	21.85	22.4	21.47	21.39	21.65
Mobuvirus alamedaense	QRW41995.1	29	17.18	17.23	17.39	17.14	17.46	18.28	21.06	21.24	21.42	22.22	20.78	21.36	20.86	22.26	22.85	22.75	20.96	21.17	20.61	20.23	21.36	20.97	21.44	23.29	21.15	21.53	20.92	20.84	100	22.31	19.12	18.59	18.67	18.75	19.02	17.21	18.55
Mobuvirus mothrae	AOF41426.1	30	18.14	18.14	18	17.71	18.03	17.98	20.63	19.42	22.22	22.61	22.31	23.23	22.14	22.61	22.62	22.3	21.51	20.7	22.16	20.05	20.51	20.64	20.84	22.11	21.58	20.69	18.25	21.06	22.31	100	18.55	18.17	17.7	18.45	19.54	18.08	17.35
Coguvirus chinense	UFE16634.1	31	20.4	20.55	21.14	19.77	20.06	20.36	21.01	19.5	21.66	21.12	22.53	22.25	20.25	21.16	21.36	21.79	21.41	21.4	20.04	19.97	21.69	21.41	21.1	20.64	20.11	20.52	19.25	23.55	19.12	18.55	100	43.29	32.04	32.26	27.98	27.64	26.97
Bocivirus gibberellae	XGD09059.1	32	20.79	20.84	20.26	19.38	19.68	20.18	20.32	19.89	21.99	20.3	22.33	22.28	19.13	20.33	22.16	22	20.96	21.86	19.86	19.74	21.93	20.88	21.9	21.34	20.2	21.03	19.36	23.23	18.59	18.17	43.29	100	30.99	31.59	29.31	27.26	26.56
Laulavirus betaviticulum	QIJ25710.1	33	19.45	19.64	18.82	18.3	19.78	18.93	20.39	20.02	20.36	19.92	21.22	22.41	19.86	20.48	21.09	21.14	20.41	20.57	20.34	20.4	19.6	19.45	18.88	19.64	18.94	19.29	20.11	21.85	18.67	17.7	32.04	30.99	100	76.31	28.54	25.56	24.88
Laulavirus alphaviticulum	QIJ25707.1	34	19.44	19.53	18.58	18.16	19.05	19.18	19.86	19.43	20.62	20.04	21.56	21.96	19.49	20.16	20.78	20.99	20.23	20.47	20.07	20.52	18.98	19.26	18.27	19.12	18.84	19.91	18.93	22.4	18.75	18.45	32.26	31.59	76.31	100	27.55	25.45	25.45
Lentinuvirus adlentinulae	QOX06031.1	35	19.13	19.27	20.37	18.71	18.43	19.8	20.38	19.52	21.08	22.04	21.65	21.73	19.88	20.78	20.24	20.49	20.95	20.63	20.01	19.29	21.24	19.93	20.3	20.98	19.02	19.61	19.71	21.47	19.02	19.54	27.98	29.31	28.54	27.55	100	25.19	24.66
Entovirus jiangsuense	QYF49543.1	36	18.26	18.39	19.05	17.82	19.15	20	19.07	17.76	20.59	20.57	20.93	20.96	18.72	20.48	19.95	19.78	19.17	18.67	18.46	18.75	19.22	19.05	18.58	20.58	18.67	18.97	18.76	21.39	17.21	18.08	27.64	27.26	25.56	25.45	25.19	100	44.98
Entovirus neimengguense	QYF49533.1	37	19.51	19.69	19.44	18.13	19.15	19.49	19.02	19	20.13	19.97	20.29	20.57	18.65	19.47	19.59	19.63	18.98	18.74	19.37	17.67	18.87	18.63	18.6	19.99	18.85	18.19	18.23	21.65	18.55	17.35	26.97	26.56	24.88	25.45	24.66	44.98	100

Figure S4. Pairwise amino acid sequence identities (%) of the L proteins between BgPWV (a1 and a2) and representative members of the family *Phenuiviridae*

Virus species	Accession	No.	1	2	3	4	5	6	7	8	9	10	11	12	13	14	15	16	17	18	19	20	21	22	23	24	25	26	27	28	29	30	31	32	33	34
Rubodvirus haikouense (isolate a2)	This study	1	100	99.21	43.75	47.04	46.9	43.97	43.4	12.75	14.55	12.65	12.06	12.24	13.53	12.1	10.22	9.55	11.7	10.79	12.39	9.8	10.11	10.67	12.43	9.54	14.11	11.02	12.68	12.98	11.49	10.73	10.38	11.88	12.88	13.21
Rubodvirus haikouense (isolate a1)	This study	2	99.21	100	43.48	47.04	46.63	43.7	43.4	12.75	14.55	12.65	12.06	12.24	13.53	12.1	10.22	9.55	11.7	10.79	12.39	9.8	10.11	10.67	12.43	9.54	14.11	11.02	12.39	12.68	11.49	10.73	10.38	11.88	12.88	13.21
Rubodvirus argentinaense	QDZ17043.1	3	43.75	43.48	100	59.89	61.23	60.7	58.13	11.73	11.61	13.95	11.63	11.5	12.14	13.11	11.29	11.11	10.32	10.89	11.3	11.7	11.7	11.42	12.01	10.51	11.21	10.58	11.05	11.34	9.13	9.13	8.78	6.44	8.99	10.86
Rubodvirus armeniaense	QDZ17040.1	4	47.04	47.04	59.89	100	83.55	67.1	61.72	11.62	11.94	14.41	12.99	12.61	10.76	12.12	11.67	11.29	9.58	10.31	11.93	10.3	11.68	11.14	12.43	11.65	12.32	9.97	13.48	14.04	10.65	10.27	9.16	10.98	12.73	15.36
Rubodvirus prosserense	AWC67528.1	5	46.9	46.63	61.23	83.55	100	69.23	62.6	10.61	11.04	13.66	12.5	11.5	11.27	12.54	10.68	10.83	10.06	10.89	12.17	11.08	11.36	11.36	11.94	11.36	11.5	9.19	12.79	12.5	8.75	8.37	9.54	10.23	13.11	14.23
Rubodvirus prosserense	AWC67529.1	6	43.97	43.7	60.7	67.1	69.23	100	60.68	12.43	11.64	14.12	11.58	12.89	12.18	11.02	9.21	9.68	10.7	11.7	12.22	12.87	13.44	12.9	13.75	9.38	12.89	10.24	12.92	13.48	10.94	10.94	9.85	11.74	12.73	13.01
Rubodvirus mal	AWC67512.1	7	43.4	43.4	58.13	61.72	62.6	60.68	100	11.05	10.98	13.8	10.67	9.97	11.33	12.47	10.05	9.65	10.36	9.97	12.46	11.89	12.16	11.62	10.99	8.73	12	11.02	10.92	10.92	9.85	10.23	8.75	10.94	11.94	13.43
Bocivirus gibberellae	XGD09060.1	8	12.75	12.75	11.73	11.62	10.61	12.43	11.05	100	34.73	32.25	31.84	32.5	34.26	32.68	28.05	26.67	24.77	25.98	25.58	26.53	26.59	26.59	25	20.15	18.87	18.6	20.15	20.39	11.99	12.36	11.65	13.43	12.18	15.5
Coguvirus rubi	WDD63193.1	9	14.55	14.55	11.61	11.94	11.04	11.64	10.98	34.73	100	48.28	42.71	47.73	44.27	40.37	25.45	24.42	28.65	23.86	26.86	23.54	23.02	23.02	25.4	16.32	18.28	17.6	20.39	21.23	13.16	13.91	13.96	11.24	10.37	14.81
Coguvirus chrysanthae	UZA34088.1	10	12.65	12.65	13.95	14.41	13.66	14.12	13.8	32.25	48.28	100	57.82	52.39	37.63	35.53	25.44	25.69	24.87	23.79	25.07	24.68	25.7	25.45	23.93	16.36	16.4	17.22	17.72	16.93	11.94	14.18	13.48	13.75	12.87	17.28
Coguvirus yunnanense	QVU28733.1	11	12.06	12.06	11.63	12.99	12.5	11.58	10.67	31.84	42.71	57.82	100	51.38	37.11	37.56	24.26	24.01	25.45	23.6	23.83	25.25	23.99	23.74	23.75	17.41	17.2	15.83	16.27	15.75	12.31	13.81	13.48	13.75	13.24	13.6
Coguvirus henanense	QVE55495.1	12	12.24	12.24	11.5	12.61	11.5	12.89	9.97	32.5	47.73	52.39	51.38	100	40.93	38.93	25.69	25.69	27.72	26.28	27.98	28.83	27.04	27.04	26.7	18.09	19.35	18.93	18.73	20.05	14.02	14.77	15.59	13.58	14.18	18.28
Entovirus neimengguense	QYF49535.1	13	13.53	13.53	12.14	10.76	11.27	12.18	11.33	34.26	44.27	37.63	37.11	40.93	100	37.69	27.48	25.95	25.97	25.71	28.91	26.09	25.32	25.06	25.77	15.79	18.82	20.05	16.54	16.01	15.09	15.09	12.88	13.16	11.9	13.06
Coguvirus chinense	UFE16635.1	14	12.1	12.1	13.11	12.12	12.54	11.02	12.47	32.68	40.37	35.53	37.56	38.93	37.69	100	21.57	21.13	26.72	26.38	28.32	27.23	25.81	25.56	24.51	14.88	17.46	20.79	17.92	17.92	12.41	12.78	12.83	10.86	11.48	11.85
Bocivirus flakkebjergense	XCO56029.1	15	10.22	10.22	11.29	11.67	10.68	9.21	10.05	28.05	25.45	25.44	24.26	25.69	27.48	21.57	100	85.56	28.51	31.84	33.56	31.39	33.54	33.33	29.94	16.25	19.35	18.58	17.92	18.4	14.18	13.82	14.6	11.68	11.19	10.36
Bocivirus viticulum	QIJ25705.1	16	9.55	9.55	11.11	11.29	10.83	9.68	9.65	26.67	24.42	25.69	24.01	25.69	25.95	21.13	85.56	100	27.89	31.69	32.72	32.45	34.44	34.22	30.92	17	19.6	18.62	18.16	18.16	13.33	13.33	13.01	11.44	11.31	9.45
Bocivirus sanyaense	UHM27567.1	17	11.7	11.7	10.32	9.58	10.06	10.7	10.36	24.77	28.65	24.87	25.45	27.72	25.97	26.72	28.51	27.89	100	62.66	58.96	49.14	48.81	49.24	47.21	16.75	19.21	18.99	17.36	17.6	11.48	10.74	9.29	10.7	8.76	11.68
Bocivirus trichodermae	WJX09057.1	18	10.79	10.79	10.89	10.31	10.89	11.7	9.97	25.98	23.86	23.79	23.6	26.28	25.71	26.38	31.84	31.69	62.66	100	62.36	53.26	53.36	53.15	49.36	18.16	19.7	19.86	20.05	19.8	11.15	10.41	9.33	10.74	8.79	11.72
Bocivirus botrytidis	QJT73692.1	19	12.39	12.39	11.3	11.93	12.17	12.22	12.46	25.58	26.86	25.07	23.83	27.98	28.91	28.32	33.56	32.72	58.96	62.36	100	52.39	54.45	54.45	49.78	18.78	21.23	20.69	20	19.51	11.19	11.19	10.86	11.9	10.29	12.5
Laulavirus gammaviticulum	QMP81964.1	20	9.8	9.8	11.7	10.3	11.08	12.87	11.89	26.53	23.54	24.68	25.25	28.83	26.09	27.23	31.39	32.45	49.14	53.26	52.39	100	54.19	53.89	52.91	19.2	21.27	19.02	18.18	18.66	13.19	12.45	12.5	12.5	11.68	13.04
Laulavirus betaviticulum	QIJ25711.1	21	10.11	10.11	11.7	11.68	11.36	13.44	12.16	26.59	23.02	25.7	23.99	27.04	25.32	25.81	33.54	34.44	48.81	53.36	54.45	54.19	100	94.34	52.76	17.46	21.32	18.47	17.35	17.59	13.55	12.45	11.03	11.03	9.85	11.59
Laulavirus alphaviticulum	QIJ25708.1	22	10.67	10.67	11.42	11.14	11.36	12.9	11.62	26.59	23.02	25.45	23.74	27.04	25.06	25.56	33.33	34.22	49.24	53.15	54.45	53.89	94.34	100	52.31	17.21	21.32	18.47	17.11	17.35	13.19	12.09	10.66	11.03	9.12	11.59
Laulavirus valsae	WNA11490.1	23	12.43	12.43	12.01	12.43	11.94	13.75	10.99	25	25.4	23.93	23.75	26.7	25.77	24.51	29.94	30.92	47.21	49.36	49.78	52.91	52.76	52.31	100	16.96	19.61	17.88	17.3	17.77	11.85	11.85	11.52	11.81	12.09	11.72
Entovirus jiangsuense	QYF49545.1	24	9.54	9.54	10.51	11.65	11.36	9.38	8.73	20.15	16.32	16.36	17.41	18.09	15.79	14.88	16.25	17	16.75	18.16	18.78	19.2	17.46	17.21	16.96	100	21.16	17.22	16.93	17.99	9.7	9.7	11.99	11.9	10.29	13.19
Lentinuvirus lentinulae	BBI93119.1	25	14.11	14.11	11.21	12.32	11.5	12.89	12	18.87	18.28	16.4	17.2	19.35	18.82	17.46	19.35	19.6	19.21	19.7	21.23	21.27	21.32	21.32	19.61	21.16	100	19.71	19.06	20.1	12.12	11.74	10.27	13.21	12.31	13.38
Lentinuvirus adlentinulae	QOX06042.1	26	11.02	11.02	10.58	9.97	9.19	10.24	11.02	18.6	17.6	17.22	15.83	18.93	20.05	20.79	18.58	18.62	18.99	19.86	20.69	19.02	18.47	18.47	17.88	17.22	19.71	100	20.38	20.14	11.48	11.11	10.78	12.92	11.31	12.36
Lentinuvirus betaarmillariae	WEA82903.1	27	12.68	12.39	11.05	13.48	12.79	12.92	10.92	20.15	20.39	17.72	16.27	18.73	16.54	17.92	17.92	18.16	17.36	20.05	20	18.18	17.35	17.11	17.3	16.93	19.06	20.38	100	89.98	12.74	13.13	12.02	12.31	12.17	13.31
Lentinuvirus alphaarmillariae	WEA82900.1	28	12.98	12.68	11.34	14.04	12.5	13.48	10.92	20.39	21.23	16.93	15.75	20.05	16.01	17.92	18.4	18.16	17.6	19.8	19.51	18.66	17.59	17.35	17.77	17.99	20.1	20.14	89.98	100	14.29	14.29	13.57	13.08	12.55	14.07
Tenuivirus festucae	QWQ49844.1	29	11.49	11.49	9.13	10.65	8.75	10.94	9.85	11.99	13.16	11.94	12.31	14.02	15.09	12.41	14.18	13.33	11.48	11.15	11.19	13.19	13.55	13.19	11.85	9.7	12.12	11.48	12.74	14.29	100	90.85	78.8	58.36	57.45	45.94
Tenuivirus persotritici	AAP82278.1	30	10.73	10.73	9.13	10.27	8.37	10.94	10.23	12.36	13.91	14.18	13.81	14.77	15.09	12.78	13.82	13.33	10.74	10.41	11.19	12.45	12.45	12.09	11.85	9.7	11.74	11.11	13.13	14.29	90.85	100	77.39	58.36	57.45	46.29
Tenuivirus oryzalbae	AUH25695.1	31	10.38	10.38	8.78	9.16	9.54	9.85	8.75	11.65	13.96	13.48	13.48	15.59	12.88	12.83	14.6	13.01	9.29	9.33	10.86	12.5	11.03	10.66	11.52	11.99	10.27	10.78	12.02	13.57	78.8	77.39	100	58.21	55.87	47.16
Tenuivirus zeae	AAB22542.2	32	11.88	11.88	6.44	10.98	10.23	11.74	10.94	13.43	11.24	13.75	13.75	13.58	13.16	10.86	11.68	11.44	10.7	10.74	11.9	12.5	11.03	11.03	11.81	11.9	13.21	12.92	12.31	13.08	58.36	58.36	58.21	100	76.68	48.94
Tenuivirus kwazuluense	WBU98207.1	33	12.88	12.88	8.99	12.73	13.11	12.73	11.94	12.18	10.37	12.87	13.24	14.18	11.9	11.48	11.19	11.31	8.76	8.79	10.29	11.68	9.85	9.12	12.09	10.29	12.31	11.31	12.17	12.55	57.45	57.45	55.87	76.68	100	47.02
Tenuivirus eurotritici	QFU20031.1	34	13.21	13.21	10.86	15.36	14.23	13.01	13.43	15.5	14.81	17.28	13.6	18.28	13.06	11.85	10.36	9.45	11.68	11.72	12.5	13.04	11.59	11.59	11.72	13.19	13.38	12.36	13.31	14.07	45.94	46.29	47.16	48.94	47.02	100

Figure S5. Pairwise amino acid sequence identities (%) of the MP proteins between BgPWV (a1 and a2) and representative members of the family *Phenuiviridae*

Virus species	Accession	No.	1	2	3	4	5	6	7	8	9	10	11	12	13	14	15	16	17	18	19	20	21	22	23	24	25	26	27	28	29	30	31	32	33	34	35	36	37	38
Rubodvirus haikouense (isolate a2)	This study	1	100	98.95	57.65	58.87	59.22	60.14	61.57	24.43	16.08	19.83	19.67	20.34	19.03	16.8	17.49	17.32	14.96	16.09	16.28	18.47	20.41	19.75	16.53	17.65	20.17	13.57	17.84	19.4	20.47	19.03	17	16.85	20.59	17.74	16.34	15.56	16.41	16.28
Rubodvirus haikouense (isolate a1)	This study	2	98.95	100	58.01	59.57	59.93	60.14	61.57	24.81	16.47	19.41	19.25	20.34	19.03	17.21	17.49	17.32	14.96	16.09	16.28	18.47	20	19.75	16.94	18.04	20.17	13.57	17.84	19.78	20.47	19.03	17	17.23	20.59	17.74	16.34	15.56	16.79	16.67
Rubodvirus argentinaense	QDZ17044.1	3	57.65	58.01	100	73.59	73.24	73.94	67.96	26.52	17.12	21.52	19.67	21.19	21.14	18.03	20.08	19.91	19.31	17.11	17.51	20.16	16.33	20.25	16.47	20.87	20.59	15.83	20.38	20.45	24	20.16	19.35	19.03	21.03	18.49	16.02	17.37	17.24	18.43
Rubodvirus armeniaense	QDZ17041.1	4	58.87	59.57	73.59	100	89.58	76.49	68.42	26.22	17.62	18.41	18.26	18.91	19.68	17.48	17.6	17.75	17.09	15.73	16.22	15.2	17.41	19.67	15.48	18.68	20.42	14.07	16.42	20.22	21.74	20.72	19.92	17.65	18.91	17.47	16.28	13.31	17.24	18.22
Rubodvirus prosserense	AWC67526.1	5	59.22	59.93	73.24	89.58	100	78.6	69.12	26.22	18.39	18.41	18.67	19.75	20.48	18.29	17.98	18.18	18.38	16.48	15.83	16	17	20.49	17.06	20.23	20	15.21	16.79	19.85	21.74	20.72	20.72	18.75	19.27	16.73	15.89	12.55	16.86	17.83
Rubodvirus prosserense	AWC67527.1	6	60.14	60.14	73.94	76.49	78.6	100	67.83	26.52	18.53	17.72	18.83	19.92	20.73	18.85	18.56	17.32	18.88	16.29	15.18	14.92	16.33	19.42	16.87	19.29	20.59	13.41	17.36	19.7	22.4	18.55	18.95	18.52	20.15	17.6	14.45	13.79	16.09	18.04
Rubodvirus mali	AWC67513.1	7	61.57	61.57	67.96	68.42	69.12	67.83	100	26.52	17.49	21.52	20.08	20.76	19.92	18.03	19.7	17.75	19.31	15.53	15.18	17.34	17.14	20.25	16.87	20.47	20.59	13.58	19.62	20.08	22.8	18.95	20.56	18.98	19.13	16.97	16.8	13.58	18.01	19.61
Fusavirus alternariae	DAZ92291.1	8	24.43	24.81	26.52	26.22	26.22	26.52	26.52	100	22.8	15.65	19.57	16.74	19.49	16.74	18.6	13.78	15.35	16.48	16.6	16.6	16.33	19.49	19.51	20.24	21.7	10.8	18.68	19.61	22.69	16.17	17.02	17.18	16.48	13.79	14.96	11.79	17.56	14.34
Hudivirus muscae	APG79300.1	9	16.08	16.47	17.12	17.62	18.39	18.53	17.49	22.8	100	15.32	16.88	18.8	24.28	18.33	22.35	18.26	19.31	23.55	21.69	20.33	22.59	20.41	23.39	26.12	20.99	19.14	17.93	17.72	17.99	19.75	21.01	14.73	15.36	16.86	22.18	16.02	18.7	18.7
Bandavirus albatrossense	AHI10997.1	10	19.83	19.41	21.52	18.41	18.41	17.72	21.52	15.65	15.32	100	36.99	39.75	32.11	30.08	28.28	23.61	22.94	27.8	21.37	21.16	19.41	18.86	19.74	23.73	17.7	17.36	15.42	17.43	18.5	21.97	20.63	17.72	16.25	21.52	20.17	10.87	13.73	15.72
Phlebovirus adanaense	AJK91621.1	11	19.67	19.25	19.67	18.26	18.67	18.83	20.08	19.57	16.88	36.99	100	54.29	35.08	36.69	28.86	24.68	22.75	26.34	19.49	21.67	25	23.68	20	20.17	20.7	18.11	19.42	18.52	17.11	20.98	21.88	16.32	19.83	25.52	18.83	16.45	15.32	16.52
Phlebovirus aguacateense	AEB70971.1	12	20.34	20.34	21.19	18.91	19.75	19.92	20.76	16.74	18.8	39.75	54.29	100	39.59	38.37	32.92	27.59	25.22	25.42	22.75	23.21	23.61	20.61	21.12	20.85	20.54	13.33	20.5	19.58	19.74	25.45	26.79	17.37	19.25	22.88	19.07	14.29	15.95	16.96
Uukuvirus rukutamaense	AII79359.1	13	19.03	19.03	21.14	19.68	20.48	20.73	19.92	19.49	24.28	32.11	35.08	39.59	100	43.31	29.02	26.05	26.47	27.02	24.28	22.76	21.9	23.48	21.99	25.21	21.83	18.07	18.73	19.05	19.92	17.17	18.88	14.92	18.73	20.56	22.41	14.71	14.05	17.87
Uukuvirus schmidti	AEL29655.1	14	16.8	17.21	18.03	17.48	18.29	18.85	18.03	16.74	18.33	30.08	36.69	38.37	43.31	100	26.98	24.47	27.23	28.57	23.55	21.54	22.73	19.13	21.01	22.5	22.27	16.26	21.37	20.08	15.38	20.43	21.3	17.96	19.35	19.59	17.84	15.32	16.6	18.1
Uukuvirus qinghaiense	XAX03433.1	15	17.49	17.49	20.08	17.6	17.98	18.56	19.7	18.6	22.35	28.28	28.86	32.92	29.02	26.98	100	21.58	23.77	24.01	24.31	20.39	20.63	18.33	17.29	20.52	20	13.9	20.65	21.1	24.7	22.76	25.2	15.55	16.79	22.11	15.43	15.12	15.2	15.48
Wenrivirus huanghaiense	QHW05230.1	16	17.32	17.32	19.91	17.75	18.18	17.32	17.75	13.78	18.26	23.61	24.68	27.59	26.05	24.47	21.58	100	62.34	23.97	24.24	21.12	19.3	20.45	19.05	22.22	18.92	18.88	17.72	23.53	24.77	19.07	18.14	15.09	19.15	16.95	18.64	14.48	17.02	17.27
Wenrivirus penaei	QPN53427.1	17	14.96	14.96	19.31	17.09	18.38	18.88	19.31	15.35	19.31	22.94	22.75	25.22	26.47	27.23	23.77	62.34	100	19.18	21.12	22.75	20.96	19	18.38	23.73	20.63	20.17	17.92	19.5	22.73	17.89	18.35	11.49	20.92	18.41	16.46	15.62	15.68	18.83
Ixovirus antarcticaense	QIS88068.1	18	16.09	16.09	17.11	15.73	16.48	16.29	15.53	16.48	23.55	27.8	26.34	25.42	27.02	28.57	24.01	23.97	19.18	100	20.7	18.73	23.89	18.44	19.7	23.22	19.42	17.24	16.76	20.19	19.51	19.09	18.26	13.67	14.78	23.32	18.62	16.6	15.08	17.74
Phasivirus alamedaense	QRW42056.1	19	16.28	16.28	17.51	16.22	15.83	15.18	15.18	16.6	21.69	21.37	19.49	22.75	24.28	23.55	24.31	24.24	21.12	20.7	100	16.73	19.92	19.33	18.37	21.03	18.72	13.06	19.61	18.5	21.49	20.76	18.64	12.94	19.07	17.9	23.32	15.79	16.8	16.26
Goukovirus ceraphri	DAZ90965.1	20	18.47	18.47	20.16	15.2	16	14.92	17.34	16.6	20.33	21.16	21.67	23.21	22.76	21.54	20.39	21.12	22.75	18.73	16.73	100	37.89	21.21	21.81	21.05	18.64	12.85	15.56	15.81	18.22	17.24	18.53	17.93	16.93	18.47	15.85	15.06	15.02	16.32
Goukovirus culicis	BEK62758.1	21	20.41	20	16.33	17.41	17	16.33	17.14	16.33	22.59	19.41	25	23.61	21.9	22.73	20.63	19.3	20.96	23.89	19.92	37.89	100	17.75	20.17	18.85	22.75	14.34	16.67	17.6	17.02	16.59	17.47	19.84	16.73	20.73	16.39	12.77	17.27	18.3
Pidchovirus pidgei	AOX47532.1	22	19.75	19.75	20.25	19.67	20.49	19.42	20.25	19.49	20.41	18.86	23.68	20.61	23.48	19.13	18.33	20.45	19	18.44	19.33	21.21	17.75	100	19.4	17.02	18.88	12.18	13.92	15.06	14.83	14.85	14.41	14.64	17.96	19.41	21.63	19.25	22.86	18.29
Hudovirus culicis	BEK62743.1	23	16.53	16.94	16.47	15.48	17.06	16.87	16.87	19.51	23.39	19.74	20	21.12	21.99	21.01	17.29	19.05	18.38	19.7	18.37	21.81	20.17	19.4	100	22.4	18.8	14.23	17.24	17.11	17.6	18.38	18.38	17.87	19.1	17.69	20.93	15.81	18.82	16.6
Citricivirus chongqinense	QDW80895.1	24	17.65	18.04	20.87	18.68	20.23	19.29	20.47	20.24	26.12	23.73	20.17	20.85	25.21	22.5	20.52	22.22	23.73	23.22	21.03	21.05	18.85	17.02	22.4	100	24.79	17.67	16.6	16.29	20	17.87	17.87	14.61	18.18	17.23	18.7	15.79	17.36	16.67
Tanzavirus daressalaamense	QDF82062.1	25	20.17	20.17	20.59	20.42	20	20.59	20.59	21.7	20.99	17.7	20.7	20.54	21.83	22.27	20	18.92	20.63	19.42	18.72	18.64	22.75	18.88	18.8	24.79	100	19.31	20.25	15.9	18.39	20	21.36	16.74	18.67	22.03	18.6	20.43	20.92	16.81
Tenuivirus eurotritici	QFU20029.1	26	13.57	13.57	15.83	14.07	15.21	13.41	13.58	10.8	19.14	17.36	18.11	13.33	18.07	16.26	13.9	18.88	20.17	17.24	13.06	12.85	14.34	12.18	14.23	17.67	19.31	100	13.73	14.01	16.12	15.83	16.25	17.33	13.14	17.05	17.6	14.23	13.55	19.23
Bocivirus gibberellae	XGD09061.1	27	17.84	17.84	20.38	16.42	16.79	17.36	19.62	18.68	17.93	15.42	19.42	20.5	18.73	21.37	20.65	17.72	17.92	16.76	19.61	15.56	16.67	13.92	17.24	16.6	20.25	13.73	100	37.82	29.18	25.4	25.4	19.64	19.43	15.6	16.12	17.44	16.46	14.92
Coguvirus chinense	UFE16636.1	28	19.4	19.78	20.45	20.22	19.85	19.7	20.08	19.61	17.72	17.43	18.52	19.58	19.05	20.08	21.1	23.53	19.5	20.19	18.5	15.81	17.6	15.06	17.11	16.29	15.9	14.01	37.82	100	30.35	25.91	26.72	21.28	23.45	17.19	16.74	15.44	17.09	15.6
Entovirus neimengguense	QYF49534.1	29	20.47	20.47	24	21.74	21.74	22.4	22.8	22.69	17.99	18.5	17.11	19.74	19.92	15.38	24.7	24.77	22.73	19.51	21.49	18.22	17.02	14.83	17.6	20	18.39	16.12	29.18	30.35	100	26.53	24.9	23.51	24.31	12.9	19.58	14.98	19.01	16.53
Laulavirus alphaviticulum	QIJ25709.1	30	19.03	19.03	20.16	20.72	20.72	18.55	18.95	16.17	19.75	21.97	20.98	25.45	17.17	20.43	22.76	19.07	17.89	19.09	20.76	17.24	16.59	14.85	18.38	17.87	20	15.83	25.4	25.91	26.53	100	74.71	23.6	21.74	20.33	14.1	17.67	17.09	19.42
Laulavirus betaviticulum	QIJ25712.1	31	17	17	19.35	19.92	20.72	18.95	20.56	17.02	21.01	20.63	21.88	26.79	18.88	21.3	25.2	18.14	18.35	18.26	18.64	18.53	17.47	14.41	18.38	17.87	21.36	16.25	25.4	26.72	24.9	74.71	100	22.8	22.92	18.7	15.38	16.87	18.38	18.6
Lentinuvirus adlentinulae	QOX06041.1	32	16.85	17.23	19.03	17.65	18.75	18.52	18.98	17.18	14.73	17.72	16.32	17.37	14.92	17.96	15.55	15.09	11.49	13.67	12.94	17.93	19.84	14.64	17.87	14.61	16.74	17.33	19.64	21.28	23.51	23.6	22.8	100	16.15	17.25	17.34	12.77	16.25	15.95
Entovirus jiangsuense	QYF49544.1	33	20.59	20.59	21.03	18.91	19.27	20.15	19.13	16.48	15.36	16.25	19.83	19.25	18.73	19.35	16.79	19.15	20.92	14.78	19.07	16.93	16.73	17.96	19.1	18.18	18.67	13.14	19.43	23.45	24.31	21.74	22.92	16.15	100	15.12	13.54	13.19	15.3	17.12
Mobuvirus mothrae	AOF41428.1	34	17.74	17.74	18.49	17.47	16.73	17.6	16.97	13.79	16.86	21.52	25.52	22.88	20.56	19.59	22.11	16.95	18.41	23.32	17.9	18.47	20.73	19.41	17.69	17.23	22.03	17.05	15.6	17.19	12.9	20.33	18.7	17.25	15.12	100	17.09	13.74	17.81	18.88
Beidivirus drosophilae	WPV74325.1	35	16.34	16.34	16.02	16.28	15.89	14.45	16.8	14.96	22.18	20.17	18.83	19.07	22.41	17.84	15.43	18.64	16.46	18.62	23.32	15.85	16.39	21.63	20.93	18.7	18.6	17.6	16.12	16.74	19.58	14.1	15.38	17.34	13.54	17.09	100	16.06	17.4	18.62
Horwuvirus fitzroyense	QLJ83472.1	36	15.56	15.56	17.37	13.31	12.55	13.79	13.58	11.79	16.02	10.87	16.45	14.29	14.71	15.32	15.12	14.48	15.62	16.6	15.79	15.06	12.77	19.25	15.81	15.79	20.43	14.23	17.44	15.44	14.98	17.67	16.87	12.77	13.19	13.74	16.06	100	14	14.87
Mobuvirus alamedaense	QRW41969.1	37	16.41	16.79	17.24	17.24	16.86	16.09	18.01	17.56	18.7	13.73	15.32	15.95	14.05	16.6	15.2	17.02	15.68	15.08	16.8	15.02	17.27	22.86	18.82	17.36	20.92	13.55	16.46	17.09	19.01	17.09	18.38	16.25	15.3	17.81	17.4	14	100	15.32
Mechlorovirus cucumeris	AYL40771.1	38	16.28	16.67	18.43	18.22	17.83	18.04	19.61	14.34	18.7	15.72	16.52	16.96	17.87	18.1	15.48	17.27	18.83	17.74	16.26	16.32	18.3	18.29	16.6	16.67	16.81	19.23	14.92	15.6	16.53	19.42	18.6	15.95	17.12	18.88	18.62	14.87	15.32	100

Figure S6. Pairwise amino acid sequence identities (%) of the N proteins between BgPWV (a1 and a2) and representative members of the family *Phenuiviridae*

Table S1. Primers for amplifying prevalence investigation of Bougainvillea progressive whitening virus (BgPWV)

Target Virus	Primer	Sequence (5' to 3')	Expected Size (bp)	Annealing Temperature (℃)	Application
BgPWV-RNA1	BgPWV-RNA1-F	TGCTTACTTACGCCTTTTTAG	639	52	Detection
	BgPWV-RNA1-R	ACTTTAGACAGGAAGAAATCGA			
BgPWV-RNA2	BgPWV-RNA2-F	CAGTAACAAAGGCACAGGA	506	54	Detection
	BgPWV-RNA2-R	TCATTGAAGCAGAAGTGACT			
BgPWV-RNA3	BgPWV-RNA3-F	TGTCTCAACTGATGCTTTCA	759	55	Detection
	BgPWV-RNA3-R	CCTTGAATGAGATCGATCGT			

Table S2. Genome organization and ORF/UTR positions of Bougainvillea progressive whitening virus (BgPWV)

Virus	RNA1			RNA2			RNA3		
	5'UTR	L	3'UTR	5'UTR	MP	3'UTR	5'UTR	N	3'UTR
BgPWV-a1	1-51	52-7851	7852-8007	1-59	59-1195	1196-1322	1-68	69-932	933-1370
BgPWV-a2	1-51	52-7851	7852-8007	1-59	59-1195	1196-1322	1-60	61-924	925-1361

All values represent nucleotide (nt) positions numbered from the 5′ terminus of each RNA segment. UTR, untranslated region; the large protein; MP, movement protein; N, nucleocapsid protein.

Table S3. Primers used to complete the genomes of Bougainvillea progressive whitening virus (BgPWV)

**Target Virus**	**Primer**	**Sequence (5' to 3')**	**Expected Size (bp)**	**Annealing Temperature (℃)**	**Application**
BgPWV-RNA1	Phen-8007-R462*	AGGACGCTTATTTAAAAGGTG	462	52	5′ terminal
	Phen-8007-F155<br>Phen-8007-R2324	ATGGCAAAGAACTTAACGGA<br>TTAAGCATTGATCCCCACTC	2170	52	complete genome
	Phen-8007-F1260<br>Phen-8007-R4043	AGGAAGCAACTTTTCAAGGA<br>AGTGAAAACCAGTGTGAGTT	2784	51	complete genome
	Phen-8007-F3697<br>Phen-8007-R5739	TCAGGGTTTATGCAAGGAAT<br>ACCTTTTATTTGCCTTCCAA	2043	52	complete genome
	Phen-8007-F5515<br>Phen-8007-R7999	TGGAGTGGAACAGGTTGTCT<br>GCCCCTCCCAATTTCATGTTG	2485	55	complete genome
	Phen--8007-F7686**	AAACGAAACATGGGGAAATG	322	56	3′ terminal
BgPWV-RNA2	Phen-1370-R264*	CTGCGTCATTCTTGTTGTTT	264	56	5′ terminal
	Phen-1370-F137<br>Phen-1370-R1296	TGTCTCAACTGATGCTTTCA<br>ATTGTCAACCGAACTCAGAA	1160	55	complete genome
	Phen-1370-F1167**	ATCAGCATGTGTTTGAAAGC	204	56	3′ terminal
BgPWV-RNA3	Phen-1322-R199*	CTCATTCAGCCTCTTTTCCT	199	58	5′ terminal
	Phen-1322-F61<br>Phen-1322-R1240	GGCTTTCTCAACAATTTGGAA<br>ACTCAGATGAAGTGATTGGG	1180	55	complete genome
	Phen-1322-F808**	AGTTGGTAGGATAAAGATAGTGACTGG	515	58	3′ terminal
	UPM	CTAATACGACTCACTATAGGGCAAGCAGTGGTATCAACGCAGAGT			

*This primer, UPM, pairs with Phen-8007-R462, Phen-1370-R264, and Phen-1322-R199 to amplify the 5' terminal sequences of RNA1, RNA2, and RNA3 of BgPWV, respectively.

**This primer, UPM, pairs with Phen--8007-F7686, Phen-1370-F1167, and Phen-1322-F808 to amplify the 5' terminal sequences of RNA1, RNA2, and RNA3 of BgPWV, respectively.
